# Transformation diffusion reconstruction of three-dimensional histology volumes from two-dimensional image stacks

**DOI:** 10.1016/j.media.2017.03.004

**Published:** 2017-05

**Authors:** Ramón Casero, Urszula Siedlecka, Elizabeth S. Jones, Lena Gruscheski, Matthew Gibb, Jürgen E. Schneider, Peter Kohl, Vicente Grau

**Affiliations:** a Institute of Biomedical Engineering, Department of Engineering Science, University of Oxford, Oxford OX3 7DQ, UK; b Heart Science Centre, National Lung and Heart Institute, Imperial College London, Harefield UB9 6JH, UK; c BHF Experimental MR Unit, Division of Cardiovascular Medicine, Wellcome Trust Centre for Human Genetics, University of Oxford, Oxford OX3 7BN, UK; d Institute for Experimental Cardiovascular Medicine, University Heart Centre Freiburg – Bad Krozingen, School of Medicine, University of Freiburg, Elsässer Str 2Q, 79110 Freiburg, Germany

**Keywords:** Diffusion equation, Translation, Similarity, Affine, B-spline transformation, Histology reconstruction, Blockface, ATDR, Approximated Transformation Diffusion Reconstruction, FTCS, Forward-Time Central-Space, TD, Transformation Diffusion, TDR, Transformation Diffusion Reconstruction

## Abstract

Traditional histology is the gold standard for tissue studies, but it is intrinsically reliant on two-dimensional (2D) images. Study of volumetric tissue samples such as whole hearts produces a stack of misaligned and distorted 2D images that need to be reconstructed to recover a congruent volume with the original sample's shape. In this paper, we develop a mathematical framework called Transformation Diffusion (TD) for stack alignment refinement as a solution to the heat diffusion equation. This general framework does not require contour segmentation, is independent of the registration method used, and is trivially parallelizable. After the first stack sweep, we also replace registration operations by operations in the space of transformations, several orders of magnitude faster and less memory-consuming. Implementing TD with operations in the space of transformations produces our Transformation Diffusion Reconstruction (TDR) algorithm, applicable to general transformations that are closed under inversion and composition. In particular, we provide formulas for translation and affine transformations. We also propose an Approximated TDR (ATDR) algorithm that extends the same principles to tensor-product B-spline transformations. Using TDR and ATDR, we reconstruct a full mouse heart at pixel size 0.92 µm × 0.92 µm, cut 10 µm thick, spaced 20 µm (84G). Our algorithms employ only local information from transformations between neighboring slices, but the TD framework allows theoretical analysis of the refinement as applying a global Gaussian low-pass filter to the unknown stack misalignments. We also show that reconstruction without an external reference produces large shape artifacts in a cardiac specimen while still optimizing slice-to-slice alignment. To overcome this problem, we use a pre-cutting blockface imaging process previously developed by our group that takes advantage of Brewster's angle and a polarizer to capture the outline of only the topmost layer of wax in the block containing embedded tissue for histological sectioning.

## Highlights

• A method for 3D reconstruction of serial 2D histology image stacks is proposed.
• Pre-alignment to an external pre-cut reference (blockface) prevents shape artifacts.
• Formulated as diffusion of transformations from each slice to its neighbors.
• Registrations replaced by much faster transformation operations.

## Graphical abstract

**Figure:**
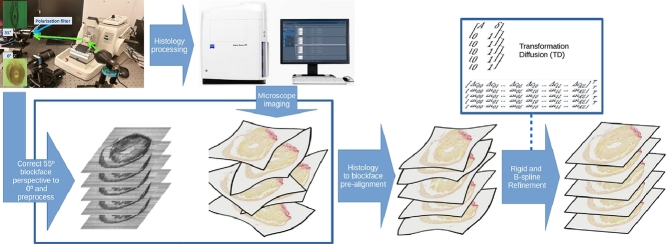

## 1 Introduction

Traditional histology, the study of tissue microarchitecture, originated in the 17th c. with first applications of microscopy to animal-derived samples by Marcello Malpighi. It has become the gold standard for structural description of cells and tissue, serving important functions in clinical diagnosis of pathologies. Traditional histology produces two-dimensional (2D) images, resolving cellular and sub-cellular detail in slices that typically are several micrometers thick. A wide variety of chromatic stains, developed since the 18th c., enable cell labeling (e.g. Masson's Trichrome or Picro Sirius Red dyes label myocytes, collagen and endothelial cells). Although most clinical tissue samples are small, typically from biopsies, interest in imaging whole organs has grown over the last decade, in organs such as brain (Amunts and Zilles, 2015, Annese, 2012), heart (Burton et al., 2006, Magee et al., 2015, Mansoori et al., 2007) or lung (Rusu et al., 2015), for instance to inform computational models that aim to simulate brain function, cardiac contraction or respiration, to guide studies relating structure to function, or to serve as a reference for lower resolution non-invasive imaging modalities such as Magnetic Resonance Imaging (Amunts et al., 2013, Plank et al., 2009, Rusu et al., 2015).

One of the main limitations of traditional histology is the fact that the acquired 2D images cannot be directly stacked to reconstruct a consistent 3D volume with the original sample shape due to a series of tissue transformations. Cardiac tissue, for example, swells by >20% during the first half-hour of ex-vivo saline perfusion (Bub et al., 2010). Histological processing for wax-embedding reduces tissue volume by 48% compared to ex vivo MRI (Burton et al., 2014), and produces non-affine deformations. Cutting of wax-embedded tissue inherently destroys the rigid alignment between 2D slices. In addition, histology stacks tend to contain large amounts of data (e.g. a rat heart, sliced at 10 µm, produces roughly 1000 slices, which, if imaged at a resolution of 0.46 µm ×  0.46 µm, require ∼1 TB hard drive space (Burton et al., 2006)). The process of recovering the sample's original 3D shape, generally referred to as *3D histology reconstruction* or *congruencing*, has received a fair amount of attention in the field since Wilhelm His’ studies of human embryos in 1880, with significant mathematical and computing improvements in the last decades.

Reconstruction of histology sections typically starts with a rough rigid pre-alignment, either registering slices to an external reference (histology-reference pre-alignment) or to each other within the stack (intra-histology pre-alignment). Pre-alignment produces jagged slice-to-slice transitions, so it is followed by finer histology registration (intra-histology refinement). Coarseness of alignment and refinement is given by the degrees of freedom of the transformation used by the registration method, e.g. rigid (Ourselin et al., 2001, Rusu et al., 2015), affine (Adler et al., 2014, Adler et al., 2012, Xu et al., 2015), 1D piecewise linear (Ju et al., 2006), elastic spring triangular mesh (Guest and Baldock, 1995, Saalfeld et al., 2012), Discrete Smooth Interpolation (Machin and Sperber, 1996), displacement field (Burton et al., 2006, Gaffling et al., 2015, Mansoori et al., 2007, Schmitt et al., 2006, Wirtz et al., 2004), curvature flow (Cifor et al., 2011, Cifor et al., 2009), symmetric normalization (SyN) diffeomorphism (Adler et al., 2012), diffeomorphic inverse consistent algorithm (Yushkevich et al., 2006), large deformation diffeomorphic metric mapping (LDDMM) (Ceritoglu et al., 2010), or tensor-product B-spline (Arganda-Carreras et al., 2010, Feuerstein et al., 2011, Gaffling et al., 2015, Magee et al., 2015, Müller et al., 2014, Roberts et al., 2012, Schubert et al., 2016, Song et al., 2013).

Algorithms that reconstruct the stack without an external reference of the pre-cut sample shape abound in the literature (Cifor et al., 2011, Fónyad et al., 2015, Gaffling et al., 2015, Guest and Baldock, 1995, Ju et al., 2006, Müller et al., 2014, Roberts et al., 2012, Saalfeld et al., 2012, Song et al., 2013, Wirtz et al., 2004, Xu et al., 2015) and are featured in software applications such as Voloom (microDimensions GmbH), BioVis3D, or 3DView (3DHISTECH Ltd.). Such reference-free approaches have long been known to be susceptible to a series of geometric artifacts. These include: “the straightening of curvatures (reconstructing a cucumber from a banana), false z-axis orientation (setting the tower of Pisa upright), or the conversion of asymmetric shapes into symmetric ones (reconstructing the bill of a raven into the bill of a woodpecker)” (Streicher et al., 1997). This set of geometric artifacts is informally known in the literature as the straight banana problem. In Section 2.3.1 we formalize this concept as the “maximum alignment” solution, and discuss its differences with the desired “true shape” solution. Other reference-free artifacts are wobbly boundaries (Ju et al., 2006) and drift or z-shift effect caused by the accumulation of correlated registration errors (Casero et al., 2016, Feuerstein et al., 2011, Yushkevich et al., 2006) (see example in Section 3.2.1). Nonetheless, reference-free reconstruction may be of interest if an external reference is simply not available, if faithful reconstruction of the shape is not crucial, or if maximum alignment coincides with the true shape, as it is the case for small rectangular or cylindrical samples with structures normal to the cutting plane. This is not the case for large cardiac samples, though, as preserving epicardial and endocardial shapes and complex structures such as locally-defined cleavage planes between myocardial layers, vasculature and trabeculae is necessary for computational modeling. Therefore, to avoid those artifacts our workflow includes an external reference, although the reconstruction algorithms we propose can be used with or without one.

Examples of external references in the literature are tissue markers (Ourselin et al., 2001, Streicher et al., 2000), drill holes (Streicher et al., 1997), template or atlas (Ali and Cohen, 1998, He et al., 1995, Ju et al., 2006, Timsari et al., 1999), structural probability map (Müller et al., 2014), MRI (Adler et al., 2014, Adler et al., 2012, Ceritoglu et al., 2010, Gibb et al., 2012, Gilbert et al., 2012, Malandain et al., 2004, Mansoori et al., 2007, Ourselin et al., 2001, Rusu et al., 2015, Schormann et al., 1995, Thompson and Toga, 1996), CT (Atkinson, 2014), micro-CT (Khimchenko et al., 2016) or 2D images of the tissue surface at the cut side of the embedded tissue, a.k.a. *blockface images* (Bardinet et al., 2002, Gefen et al., 2003, Kim et al., 1997, Mega et al., 1997, Ourselin et al., 2001, Schubert et al., 2016, Siedlecka et al., 2013a, Siedlecka et al., 2013b, Toga et al., 1994). Taking a different approach, (Xu et al., 2015) use bisected nuclei in liver histology as natural fiducial markers to avoid geometric artifacts without an external reference. This requires a sufficiently uniform distribution of bisected nuclei, which is not guaranteed for cardiac tissue, in particular in areas where myocytes run orthogonal to the cutting plane. Also, nuclei visualization limits the number of dyes that can be used. Our external reference is a novel type of blockface image developed by our group (Casero et al., 2016, Gruscheski et al., 2015, Siedlecka et al., 2013a, Siedlecka et al., 2013b). Our method takes advantage of light polarization when illuminating the wax top surface at Brewster's angle to produce a sharp near-binary ‘negative’ image of the regions where tissue protrudes. Unlike 3D images obtained prior to histological processing, such as CT or MRI, blockface images are acquired directly at the microtome and do not involve an ill-posed 2D→3D alignment problem caused by different slicing angle between histology and the 3D image, as well as 3D tissue deformations out of the slice plane, as seen in previous work by our group (Gibb et al., 2012, Mansoori et al., 2007). Furthermore, the 2D→2D alignment problem is trivially parallelizable. In common with the majority of the literature, we only use the blockface images to pre-align the histology stack. Alternatively, (Adler et al., 2014, Adler et al., 2012, Feuerstein et al., 2011, Mansoori et al., 2007) use the external reference during refinement. In this case, the external reference can be seen as a regularization term that also introduces registration noise, caused by its lower resolution and imaging artifacts, and interferes with the delicate local transformations necessary to align small structures. Another alternative is to first refine the histology stack and then register to an external MRI reference solving a 3D→3D alignment problem (Ceritoglu et al., 2010, Malandain et al., 2004). For the blockface external reference, this approach would need to be adapted as a regularized 2D→2D alignment to take advantage of the blockface-histology slice-by-slice correspondence, and is beyond the scope of this work.

Apart from the type of registration method and the use of an external reference, another main feature of reconstruction methods is how they sweep the stack of *N* histology slices $I_{0},\ldots ,I_{N-1}$. The prevalent approaches in the literature are sequential algorithms that register one slice at a time towards one or more neighbors, applying the resulting transformation straight away. Any slice can be used as the initial one, but to simplify the notation, let's assume that the sweep starts at *I*_0_. Algorithms that register each slice *I_i_* to a unilateral radius-*d* neighborhood $I_{i-d},\ldots ,I_{i-1}$ need only one sweep of the stack to converge because they are causal (Arganda-Carreras et al., 2010, Burton et al., 2006, Ourselin et al., 2001, Roberts et al., 2012, Rusu et al., 2015, Schmitt et al., 2006, Schubert et al., 2016, Song et al., 2013, Xu et al., 2015). The methods in Adler et al., 2014, Adler et al., 2012), Yushkevich et al. (2006) are also causal sequential, but the neighbor $I_{i-k}$ that *I_i_* is registered to is not necessarily $I_{i-1}$. Non-causal algorithms that align *I_i_* to a bilateral neighborhood $I_{i-d},\ldots ,I_{i-1},I_{i+1},\ldots ,I_{i+d}$ (Gaffling et al., 2015, Guest and Baldock, 1995, Saalfeld et al., 2012, Wirtz et al., 2004) cannot converge in one sweep, because after *I_i_* is registered to $I_{i+1},\ldots ,I_{i+d}$, those neighbors get transformed too. Instead, repeat sweeps (forwards or back and forth) can be used to increasingly smooth the stack, but this increases the computational cost, particularly if the algorithm depends on expensive registration operations. However, whether causal or non-causal, sequential methods are biased towards the choice of the reference slice, and correlated registration errors accumulate along the sweep, causing drift (Yushkevich et al., 2006). Smoothing accumulates along the sweep too, as slice *I_i_* is registered to previously smoothed slices $I_{i-d},\ldots ,I_{i-1}$.

Parallel algorithms avoid bias, reduce drift and apply uniform smoothing by computing all local neighborhood transformation $\phi_{i,i-d},\ldots ,\phi_{i,i-1},\phi_{i,i+1},\ldots ,\phi_{i,i+d}$, where *ϕ*_*i, j*_ is the transformation from *I_i_* to *I_j_*, before applying any slice transformation *ϕ_i_*. Parallel algorithms that couple all $\phi_{i,j},i=0,\ldots ,N-1,\;j=i-d,\ldots ,i+d$ into a system of equations require one registration stack sweep followed by solving the system. For instance, (Guest and Baldock, 1995, Saalfeld et al., 2012) use an elastic spring Finite Element Model for the system of equations. The whole stack system is considered intractable, so the reconstruction is solved as a non-causal sequential sweep. Feuerstein et al. (2011), Müller et al. (2014) propose a Markov Random Field model on B-spline control points that can be solved for the whole stack with a Linear Programming method (Glocker et al., 2008), but it allows only a discrete set of locations the control points can move to. Parallelization of the refinement involves parallelization of the Linear Program primal-dual schema solver.

As an alternative to solving a large system of equations, uncoupled parallel methods update each slice independently, $\phi_{i}=f\left(\phi_{i,i-d},\ldots ,\phi_{i,i-1},\phi_{i,i+1},\ldots ,\phi_{i,i+d}\right)$ (Ju et al., 2006). Thus, they are non-causal. If one sweep does not achieve enough smoothing, the algorithm can be applied iteratively, increasing its computational cost. (Most sequential methods can also be run as uncoupled parallel algorithms with minimal modifications, trading their advantages and drawbacks.) The algorithms we propose in this work are uncoupled parallel, but we show that replacing registration operations by operations in transformation space, they have same computational load as causal sequential algorithms.

Another main feature of reconstruction methods is the neighborhood, already mentioned above. Some authors propose radius-1 neighborhoods, where each slice is only registered to adjacent slices (Arganda-Carreras et al., 2010, Gaffling et al., 2015, Roberts et al., 2012, Song et al., 2013, Stille et al., 2013), whereas other authors propose larger neighborhoods. Typically, larger neighborhoods are used to increase smoothing in each sweep, thus reducing the amount of sweeps (Ju et al., 2006), or as a regularizer to avoid overcorrection (Saalfeld et al., 2012). However, Gaffling et al. (2015) showed that radius-1 neighborhoods can effectively avoid overcorrection, removing high frequency registration noise before affecting the lower frequencies of the specimen's shape. Yushkevich et al. (2006) use a larger neighborhood to find a subsampling of the stack where slices are better aligned. As their radius-1 sequential alignment effectively uses a shorter stack per slice, this would accumulate fewer correlated errors, but this approach still produces substantial reconstruction artifacts that need to be corrected with an external reference. Typical larger neighborhoods can be implemented as linear combinations of transformations between pairs of slices (Ju et al., 2006, Rusu et al., 2015), or connecting elastic springs between non-adjacent slices (Guest and Baldock, 1995, Saalfeld et al., 2012). Overall, this choice as well as parameters such as radius or weights remains heuristic. Our algorithms in this work use a radius-1 neighborhood in the implementation, but we show its equivalence to a larger neighborhood in terms of smoothing.

Most methods in the literature are based on image registration between pairs or images, but some alternatives have been proposed. For instance, Machin and Sperber (1996) build a rectangular mesh with segmented contours, and sequentially smooth the mesh vertices with Discrete Smooth Interpolation. Cifor et al., 2011, Cifor et al., 2009) also stack segmented contours but smooth the curvature of the resulting surface with a level set algorithm. Level sets can be parallelized, but both approaches require reliable segmentation of stackable contours for every structure to be aligned.

In this paper we propose a mathematical framework for histology reconstruction called *Transformation Diffusion (TD)* that tackles several of the limitations of the methods we discuss above. We model stack misalignment applying the heat diffusion equation to slice transformations. TD is agnostic of the registration method employed, and only depends on the transformation it produces. Our solution to the heat equation produces a simple update formula with a radius-1 neighborhood that is trivially parallelizable. We also propose replacing registrations by operations in transformation space that are several orders of magnitude faster. Effectively, our method computes only (N−1) registrations in the first stack sweep, and the cost of other sweeps is negligible. Combining these ideas, we propose a general algorithm called *Transformation Diffusion Reconstruction (TDR)* that is valid for transformations that are closed under inversion and composition, and provide specific formulas for the cases of translation and affine transformations. In addition, for tensor-product B-splines, which are not closed under inversion and composition, we propose an *Approximated Transformation Diffusion Reconstruction (ATDR)* algorithm. This algorithm applies TDR to the spline's control polygon, and uses constraints to guarantee injectivity. ATDR computes 2(N−1) registrations in the first stack sweep, and the cost of other sweeps is negligible.

TD provides some insights into the reconstruction problem. We discuss a formal definition of the banana problem and the desired true shape reconstruction, the role of the external reference, the equivalence of TDR/ATDR to a global Gaussian low-pass filter, and the equivalence between neighborhood radius and number of stack sweeps. The algorithms only depend on two parameters, for which we provide theoretical and numerical analyses: the diffusion step *α* and the number of stack sweeps *M*, a simple stopping criterion that determines the bandwidth of the implicit Gaussian filter.

For comparison, two approaches have a close connection to ours. Gaffling et al. (2015) pose the problem of histology smoothing as intensity curvature minimization using Laplace's partial differential equation (PDE)

(1) $$0=\nabla^{2}I\left(r\right),$$

where ∇^2^ is the Laplacian along stack direction *r*. Discretization of this equation produces a sequential algorithm with a radius-1 neighborhood where back and forth sweeps increasingly smooth the stack. Using the relationship between spatial frequency and eigenvector decomposition of Jacobi iteration matrices, they notably provide a theoretical model that explains how registration noise is smoothed more than lower frequency anatomical structures in their algorithm. Their approach is non-causal sequential, suffering from the drawbacks discussed above. In addition, the model leads to a Gauss–Seidel algorithm that updates *I_i_* using $I_{i-1},\;I_{i+1}$ without *I_i_* itself. This is solved with an ad hoc approximation, registering *I_i_* to $I_{i-1}\circ \frac{1}{2}\phi_{i-1,i+1}$; moreover, this definition of the mid-point of the transformation is inexact in general (Alexa, 2002). By comparison, our approach is also based on the discretization of a similar PDE, but it leads to a parallel algorithm where the space of operations is well-defined (Alexa, 2002), and explains spatial frequency smoothing as filtering with a Gaussian function. Yushkevich et al. (2006) propose pre-aligning the histology to an external reference, and then replacing the costly intra-histology registration sweeps by much faster operations in the space of transformations. However, they are limited to rigid transformations, and they smooth the transformations by applying a Gaussian filter directly to their parameters. The latter is equivalent to a linear combination of rigid parameters and produces poor interpolations for rotations (Alexa, 2002). Our approach also replaces registrations by operations in transformation space, but we provide expressions that are exact for the spaces of translation and affine transformations, and an approximation for B-spline transformations. Relatedly, the field of multi-atlas segmentation literature offers a variety of transformation-space registration approaches (Iglesias and Sabuncu, 2015).

This paper is organized as follows. In Section 2.1 we describe the wet lab processing of mouse hearts, and blockface and histology imaging. In Section 2.2, we briefly describe the image preprocessing pipeline and the registration methods used in the experiments. In Section 2.3, we formulate Transformation Diffusion (TD). In Section 2.4 we describe the Transformation Diffusion Reconstruction (TDR) algorithm, and derive formulas for translation and affine transformations. In Section 2.5 we describe the Approximated TDR (ATDR) algorithm for tensor-product B-splines. Section 3 validates experimentally the application of TDR and ATDR. First, we validate TDR with a noisy sinusoidal example for translation transformations, and compare it to Gaffling's Gauss–Seidel scheme. Second, we validate TDR/ATDR with real data with the reconstruction of a mouse heart, with and without an external reference. We also compare ATDR to a baseline algorithm that performs repeat registration sweeps, accumulating levels of B-spline transformations. We discuss our results in Section 4. Appendix A provides a theoretical and numerical analysis of diffusion step parameter *α*. Appendix B shows the equivalence of the radius-1 neighborhood of TD to a larger neighborhood. Appendix C provides the injectivity constraints for B-spline transformations in ATDR. The blockface and histology images can be downloaded from (Siedlecka et al., 2017). The source code to run the experiments and generate the figures in this paper is available from the Gerardus project.^1^ The histology reconstructed with our method can be downloaded from (Casero et al. 2017).

## 2 Methods

### 2.1 Wet lab processing

Mouse hearts were excised after Schedule 1 culling according to the UK Home Office guidance on the Operation of Animals (Scientific Procedures) Act of 1986, washed swiftly in 37 °C normal Tyrode solution (NaCl 140 mM; KCl 5.4 mM; MgCl_2_ 1 mM; HEPES 5 mM; Glucose 10 mM; CaCl_2_ 1.8 mM; pH 7.4, 300 ± 10 mOsm) containing heparin (10 u/ml) and then cannulated in cold Tyrode solution with heparin in order to perfuse the coronary vasculature from the aorta. Afterwards, hearts were arrested using modified Tyrode containing elevated potassium (20 mM), fixed by coronary perfusion with 10 mL of the fast-acting Karnovsky's fixative^2^ (0.75% formaldehyde, 0.75% glutaraldehyde mix) and stored overnight at 4 °C. Hearts then were rinsed in cacodylate buffer (3×), dehydrated by exposure to rising alcohol concentrations (8 h in 25%, followed by 1.5 h in each of 50/70/80/90% alcohol, and 3 × 1.5 h in 100% alcohol), treated with xylene (3 × 1.5 h), infiltrated with wax (24 h in 50% and 48 h in 100%) and embedded in form of wax blocks. Wax blocks were mounted on Leica SM2400 heavy-duty sledge-type microtome and whole hearts were serially sectioned at 10 µm thickness.

**Fig. 1:**
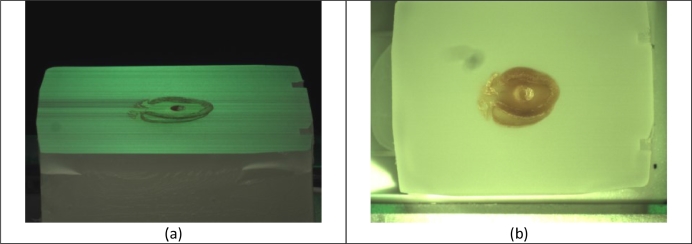
Two blockface photographs of the same wax-embedded mouse heart. (a) Brewster angle (55°) image with the collection filter aligned to capture surface-parallel polarized light, reflected by the topmost wax layer, revealing tissue outlines (centrally-located round mark is an air bubble in the wax). (b) Top-down (0°) image, taken for distortion correction, lacks clear delineation of tissue-wax boundaries.

Using two The Imaging Source DMK 22BUC03 cameras, two photographs of the wax block surface were taken prior to each section (Fig. 1), an approach developed by our group in the last years (Casero et al., 2016, Gruscheski et al., 2015, Siedlecka et al., 2013a, Siedlecka et al., 2013b). The first photograph was taken at the Brewster angle for air/paraffin (55° to the surface normal). At this angle, the surface-vertical component of unpolarised incident light is totally transmitted, while most of the surface-horizontal component is reflected. A collection filter, aligned with the horizontal component filters out residual light refracted from lower tissue layers. Thus, the 55° image treats wax as a mirror and displays a negative of the topmost tissue layer, showing good delineation of tissue-wax boundaries. The second photograph was taken top-down (0° to the surface normal) as a reference to correct the perspective distortion in the 55° image (see Section 2.2).

After cutting, tissue sections were allowed to relax in a water bath (Leica Microsystems, HI 1210) at 39 °C for several minutes, and then carefully mounted on positively charged slides (SuperFrost, VWR), aiming for minimal distortion and avoidance of tissue folds. Slides were air-dried overnight, followed by de-waxing and Trichrome or Picro Sirius Red-staining on alternate sections, using a Leica AutoStainer XL, ST5010. The stained and coverslipped sections were imaged at high resolution (0.46 µm × 0.46 µm) with Hamamatsu Nanozoomer HT 2.0 and/or Zeiss Axio Scan.Z1 scanners, producing images between 8862 × 6643 (86 M) and 28,672 × 23,296 pixels (1.2G) depending on tissue sample size.

### 2.2 Image preprocessing and registration methods used in the experiments

**Fig. 2:**
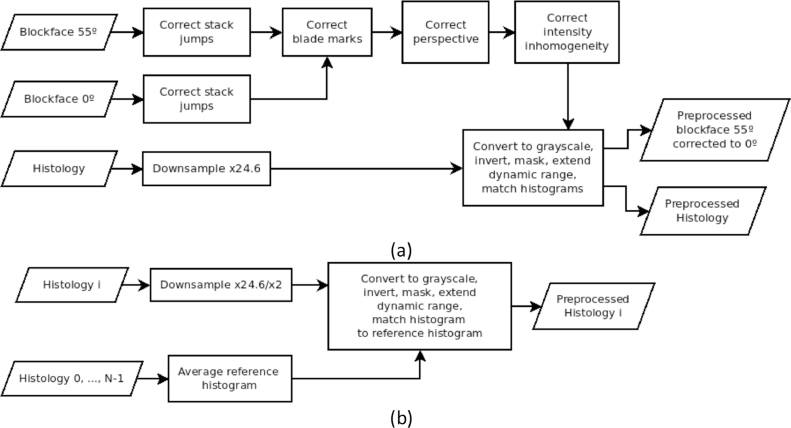
Diagram of blockface and histology preprocessing pipeline for: (a) low resolution histology-to-blockface pre-alignment; (b) histology-to-histology refinement, run separately for low and high resolution histology, where “Downsample × 24.6/× 2” indicates × 24.6 downsampling to blockface pixel size for low resolution refinement, and ×2 downsampling for high resolution refinement. See main text for details.

The algorithms we propose in this paper, TDR and ATDR, are independent of the image preprocessing pipeline or registration method details, other than the type of transformation. The former are often specific to the image modality and protocol, but we provide a brief summary in this section for reproducibility of our results. Blockface and histology images suffer from a range of artifacts such as non-uniform illumination, blade marks in the blockface (“scratches”), 55° perspective distortion (for Brewster angle images), or folds, tears, and variable staining of sections. Adjustments to the equipment while developing the wet lab protocol caused small blockface “jumps” due to changes in the light pathways, e.g. minute shifts of a lens, the camera, the microtome and/or zoom-in or zoom-out changes. In addition, the second half of the heart was cut at a much later date, rotated 90° with respect to the first half, causing a large “jump” in the stack. Such “jump” artifacts would not be expected in a production run, and were corrected. The rest of artifacts were corrected as we would in a production run, to make the images as similar as possible and enable the use of computationally faster metrics such as mean squares. We applied the following preprocessing steps before histology-to-blockface registration (Fig. 2a): (1) Stack “jumps” were visually identified, and automatically corrected with a similarity transformation. (2) We corrected blade scratches highlighted by the polarized light in the perspective-corrected 55° image by estimating the median angle *α* to the wax side and rotating the images to make the scratches horizontal/vertical for the first/second halves of the heart. We then scaled the intensity along each row/column (i.e. scratch) so that its median value equaled the median wax intensity. (3) We corrected the perspective of the 55° image with a projective transformation (Hartley and Zisserman, 2003) computed from 82 pairs of hand-traced landmarks in 11 equidistantly spaced slices of both stacks. (4) To correct the blockface illumination inhomogeneity we drew an ellipse covering the heart area on the largest slice; in every slice, the illumination within the ellipse was interpolated from the intensity values outside the ellipse with a 2D polynomial function of degree 5, as this interpolant worked well enough for our data. Dividing by the estimate resulted in uniform illumination. (5) The histology images were × 24.6 downsampled in each axis to blockface pixel size for the low-resolution reconstruction. (6) The histology and blockface images were converted to grayscale, inverted to generate a negative of the image, and masked to remove background noise. Blockface intensities were linearly scaled to cover the whole dynamic range (0–255), and then histology intensities were non-linearly scaled (Ceritoglu et al., 2010) to match the blockface image histogram. Matching histograms improves the performance of registration based on intensity differences. Similarly, we could have matched the blockface histograms to the histology's.

Before histology-to-histology refinement, we applied the following preprocessing steps (Fig. 2b): (1) Images were × 24.6 downsampled in each axis to blockface pixel size for the low-resolution refinement, and ×2 downsampled for the high-resolution refinement. (2) Similarly to step 6) above, each image was converted to grayscale, inverted, and masked. Then, intensities were linearly scaled to cover the whole dynamic range (0–255) and non-linearly scaled (Ceritoglu et al., 2010) to match an average histogram computed from all slices.

For the image registration steps in our reconstruction methods we used similarity (for intra-blockface) and rigid and cubic B-spline (for histology-blockface and intra-histology) transformations. For rigid registration we used a matched filter approach to find the translation parameters (Moigne et al., 2011). The optimal rotation was found by brute force in increments of 1° between −45° and 45°. As our diffusion framework is independent of the particular registration method, it was beyond the scope of this paper to conduct extensive comparisons of different methods.

For similarity and cubic B-spline registration we used the elastix software (Klein et al., 2010, Shamonin et al., 2014), selecting a mean squared difference metric, and adaptive stochastic gradient descent optimization. For similarity registration we used cubic interpolation and a single resolution. For low resolution B-spline registration, bilinear interpolation, fixed boundary control points, two-level Gaussian pyramid scheme, ×16 and ×1 downsampling for both the image and control polygon grid, and final control point spacing of 50 pixels. This provided good low resolution alignment while keeping registration times around 1–2 s for each pair of slices. For high resolution B-spline registration, we changed the Gaussian pyramid scheme to ×8 and ×4 downsampling for the image and ×4 and ×1 for the control polygon, with final control point spacing of 35 pixels. This roughly corresponds to the distance between myocytes separated by a cleavage plane, which is the finest level of microstructure we are interested to align.

### 2.3 Transformation diffusion (TD)

This section presents the mathematical framework that is one of the main contributions of this paper. TD iteratively takes at the input a set of geometric transformations (e.g. translation, affine) between pairs of adjacent slices. At every iteration, it *diffuses* these slice-to-slice transformations to partly deform each slice towards its neighbors. TD is based on an iterative numerical solution to the heat equation, and replaces slow registrations by much faster operations in transformation space.

#### 2.3.1 The reconstruction problem: maximum alignment solution vs. desired true shape solution

**Fig. 3:**
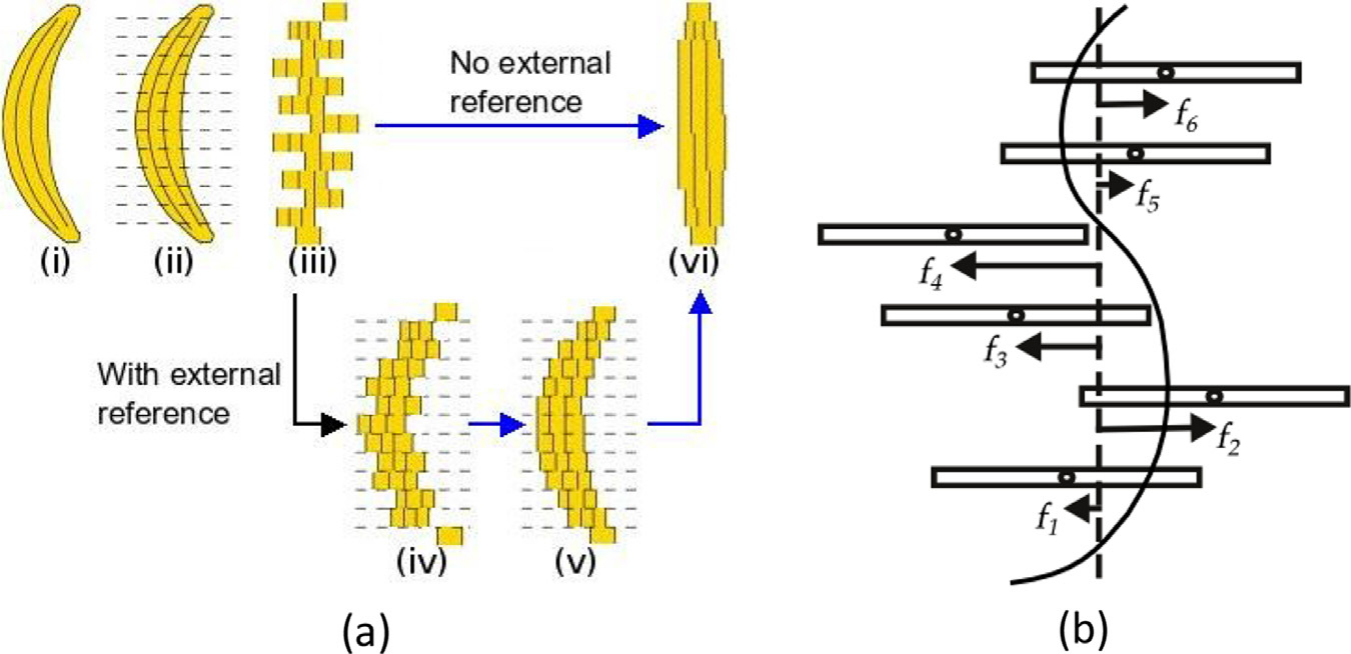
(a) Banana problem (expanded from Malandain et al., 2004 with permission): (i) Original banana specimen. (ii) Microtome slicing. (iii) Initial stack of histology images, where misalignment can be seen as transformation noise. (iv) Noisy alignment to external reference. (v) Desired true shape histology or curved banana solution. (vi) Maximum alignment or straight banana solution, i.e. limit of refinement/smoothing of the stack. Black arrow indicates histology-blockface alignment. Blue arrows indicate intra-histology refinement. (b) Schematic representation of a set of unregistered histology slices. The solid curve represents the desired true shape histology solution. The dashed line represents the “maximum alignment” solution between all slices that intra-slice refinement algorithms (including our Transformation Diffusion method) tend to in the limit, analogous to the thermal equilibrium solution. Each *f_i_* is the transformation of slice *i* referred to maximum alignment, unknown in reconstruction problems. Although *f_i_* is represented as a simple horizontal translation, in general it is a non-affine transformation with multiple degrees of freedom. (For interpretation of the references to colour in this figure legend, the reader is referred to the web version of this article.)

The banana problem is well known in the reconstruction literature (Fig. 3a). Let $I_{0},\ldots ,I_{N-1}$ be a stack of 2D histology slices, pre-aligned or not to an external reference, and $f_{0},\ldots ,f_{N-1},f_{i}$: ℝ^2^ ↦ ℝ^2^ the current transformation of each slice away from *maximum alignment* (Fig. 3b). Let maximum alignment be a configuration where the alignment between adjacent slices cannot be improved. Formally, if all $f_{i}=0$, then $f_{i,i-1}=f_{i,i+1}=0,\;\forall \;i$, where *f*_*i, j*_ is the transformation that registers slice *i* to slice *j*. The maximum alignment solution varies with the type of transformation. For instance, the maximum alignment solution for translation transformations is the straight banana in Fig. 3a–vi. For similarity transformations, however, it is a cylindrical banana where all slices have the same radius. The relation between the initial stack, the desired solution and the maximum alignment solution is displayed in Fig. 3a. The solution depends on whether an external reference is used, and the amount of refinement/smoothing applied to the stack is determined by a combination of empirical stopping criteria, algorithm parameters and/or regularizers.

For example, Gaffling et al. (2015) smooth the stack *M* times, stopping when the update of the solution reaches an empirical threshold, or by visual assessment (M=10 sweeps for their mouse brain experiment). Cifor et al. (2009) use an empirical number of smoothing steps, and control the amount of smoothing with the flow speed constant *b_k_* (M=153, $b_{k}=0.05$ in their experiments). Cifor et al. (2011) replace those stopping criteria by an empirical min-max curvature flow scale parameter (r=1 and r=2 in their experiments). Ju et al. (2006), Saalfeld et al. (2012) sweep the stack once. This is itself an implicit criterion (M=1), as more sweeps would produce further smoothing. The amount of smoothing in the sweep is determined by empirical parameters. For Saalfeld et al.'s (2012) elastic spring system, the parameters are the size of the neighborhood and the value of spring constants (6 neighbors, k=0.1 in their experiments). This model tends to maximum alignment if the intra-slice spring constants *k* → 0 and the neighborhood is the whole stack. The empirical parameters for Ju et al. (2006) are the number of neighbors and the weights *γ_i_* for the linear combination of transformations (40 neighbors and binomial *γ_i_* in their mouse brain experiment). Adler et al. (2014), Rusu et al. (2015) use a regularization framework, minimizing a function $\lambda E_{in}+\mu \;E_{ext}$, where *E_in_* is a measure of intra-histology misalignment, *E_ext_* is a measure of histology to external reference misalignment, and *λ, μ* are empirical parameters that decide a compromise between the straight banana (*E_in_*) and noisy banana (*E_ext_*) solutions.

In this work we use *M* to regulate the amount of smoothing. We provide a detailed discussion in Section 2.3.6.

#### 2.3.2 Heat equation formulation for translation refinement

We pose the reconstruction problem in a heat diffusion framework. A discretized heat diffusion problem can be thought of as a set of points $I_{0},\ldots ,I_{N-1}$ along a metal bar and $f_{0},\ldots ,f_{N-1}$ as their temperature difference with respect to thermal equilibrium rather than their absolute temperature. As time increases, *f_i_* → 0, and the bar reaches thermal equilibrium. By gross analogy, in the stack refinement problem (Fig. 3b) the transformations *f_i_* → 0 as time increases, and the stack reaches maximum alignment. An important difference is that in a heat diffusion problem the initial values of $f_{0},\ldots ,f_{N-1}$ are usually known, whereas in a stack refinement problem they are unknown by definition. In the rest of this section we present the mathematical formulation of this idea with a simple case of a 1-dimensional translation transformationf=ϕ, where changes in temperature are analogous to translations.

The heat equation (Narasimhan, 1999) describes the change of *ϕ* over time 0 ≤ *t* < ∞ and position 0 ≤ *r* ≤ *L* for a continuous stack with an infinite number of infinitesimally close slices

(2) $$\frac{\partial \phi \left(r,t\right)}{\partial t}=D\nabla^{2}\phi \left(r,t\right),$$

where ∇=∂/∂r, and constant scalar *D* is the diffusion coefficient. If the initial condition *ϕ*(*r*, 0) is known, the general solution to the heat equation can be obtained explicitly by convolution ⋆

(3) ϕ(r,t)=ϕ(r,0)☆G(r,t),

where *G* is the Gaussian kernel

(4) $$G\left(r,t\right)=\frac{1}{{\left(4\pi Dt\right)}^{1/2}}\exp\left(-\frac{r^{2}}{4Dt}\right).$$

As *t* → ∞, $\phi \left(r,t\right)\to \bar{\phi }$, where $\bar{\phi }$ is the average function value, equilibrium or maximum alignment. As the number of slices is finite, we discretize the heat equation. For this, we use a standard approach. For the N−2 inner slices, using the Forward-Time Central-Space (FTCS) method (Roache, 1972) produces

(5) $$\frac{\phi_{i}^{m+1}-\phi_{i}^{m}}{\Delta t}=D\frac{\phi_{i+1}^{m}-2\phi_{i}^{m}+\phi_{i-1}^{m}}{\Delta s^{2}},\;i=1,\ldots ,N-2,$$

with *m* denoting the iteration number, *Δs* the spatial increment, *Δt* the time increment, and $\phi_{i}^{m}=\phi \left(i\;\Delta s,m\;\Delta t\right)$. For the two end slices, i=0 and i=N−1, we impose Neumann boundary conditions as Gaffling et al. (2015), as these do not fix their position, which is convenient for reconstruction

(6) $$\begin{array}{c} \nabla \phi \left(0,t\right)=0\\ \nabla \phi \left(L,t\right)=0 \end{array}$$

To match the $O(\Delta s^{2})$ truncation error in (5), we discretize the boundary conditions with centerd differences (Pletcher et al., 2013)

(7) $$\begin{array}{c} \phi_{-1}^{m}=\phi_{1}^{m}\\ \phi_{N-2}^{m}=\phi_{N}^{m} \end{array}$$

where $\phi_{-1},\phi_{N}$ correspond to “ghost” slices outside the stack. Solving (5) for $\phi_{i}^{m+1}$ with (7) produces an iterative update formula that in the limit *m* → ∞ tends to the discretized version of the solution in (3). But note that this approach requires knowledge of the (discretized) initial condition $\phi_{i}^{0}$. As by definition the slice deformations $\phi_{i}^{0}$ are unknown, we depart from the conventional solution and reformulate (5) in terms of known quantities, namely transformations between adjacent slices. Let

(8) $$\phi_{i,j}^{m}=\phi_{j}^{m}-\phi_{i}^{m}$$

be the translation difference between two neighboring locations at the same iteration, and let

(9) $$\phi_{i}^{m,m+1}=\phi_{i}^{m+1}-\phi_{i}^{m}$$

be the translation difference at the same location between two iterations. Now (5) and (7) can be rewritten and combined into the update formula

(10) $$\phi_{i}^{m,m+1}=\left\{ \begin{array}{cc} 2\alpha \phi_{0,1}^{m}, & i=0\\ \alpha \left(\phi_{i,i+1}^{m}+\phi_{i,i-1}^{m}\right), & i=1,\ldots ,N-2\\ 2\alpha \phi_{N-1,N-2}^{m}, & i=N-1 \end{array}\right.$$

Where the diffusion step $\alpha =D\;\Delta t/\Delta s^{2}$. Note that this expression, unlike more commonly used iterative solutions to the discretized heat equation, does not require knowledge of $\phi_{i}^{0}$, as it only depends on local translations $\phi_{i,j}^{m}$ between adjacent slices. In (10), the transformation to apply to a slice is a linear combination of the transformations that align it to its neighbors. As *M* → ∞, the composition of the transformation updates tends to the inverse of the slice's unknown initial misalignment, $\left(\phi_{i}^{M-1,M}\circ \ldots \circ \phi_{i}^{0,1}\right)\to \phi_{i}^{-1}$.

From theory, it is known that FTCS is numerically stable if and only if 0 ≤ *α* ≤ 0.5 (Pletcher et al., 2013). As an intuitive explanation, with α=0.5 each slice deforms half the distance to its neighbors, so neighbors try to meet in the middle; with *α* > 0.5 the slices deform past each other, producing oscillations; with *α* < 0.5 neighbors deform towards each other, but fall short of the middle point. In principle, larger diffusion steps *α* required fewer stack sweeps. However, in Appendix A we show that values of *α* too close to 0.5 do not dampen high frequency noise, and thus we use α=0.45 in the experiments below.

To close this section, it is worth noting that, strictly speaking, the above analysis only applies to translation transformations. In the next sections, we assume that the formulation can be applied to more general transformations too, and hope to extend the relevant analysis in future work.

#### 2.3.3 Generalization of update slice transformation step

To apply the transformation update in (10) to real-world stack reconstruction, we need to generalize 1D translations *ϕ* and the product and sum operators. We generalize the sum of translations in (8) as a composition of n-D invertible transformations

(11) $$f_{i,j}^{m}=f_{j}^{m}\circ {\left(f_{i}^{m}\right)}^{-1}.$$

Furthermore, following Alexa (2002) we generalize the linear combination of two translations as $\alpha ⊙(f_{i}^{m}⊕f_{j}^{m})$, where ⊙, ⊕ are generalized scalar product and addition operators consistent with the transformation *f* (see Sections 2.4.1 and 2.4.2 for specific examples of the operators), since simple linear combination of transformations $\alpha (f_{i}^{m}+f_{j}^{m})$ does not produce a valid interpolation of transformations in general. Applying these generalizations to (10) we obtain the *update slice transformation step*

(12) $$f_{i}^{m,m+1}=\left\{ \begin{array}{cc} \alpha ⊙\left(f_{0,1}^{m}⊕f_{0,1}^{m}\right), & i=0\\ \alpha ⊙\left(f_{i,i+1}^{m}⊕f_{i,i-1}^{m}\right), & i=1,\ldots ,N-2\\ \alpha ⊙\left(f_{N-1,N-2}^{m}⊕f_{N-1,N-2}^{m}\right), & i=N-1 \end{array}\right.$$

If ⊕, ⊙ fulfill the distributive property, this expression simplifies to

(13) $$f_{i}^{m,m+1}=\left\{ \begin{array}{cc} 2\alpha ⊙f_{0,1}^{m}, & i=0\\ \alpha ⊙\left(f_{i,i+1}^{m}⊕f_{i,i-1}^{m}\right), & i=1,\ldots ,N-2\\ 2\alpha ⊙f_{N-1,N-2}^{m}, & i=N-1 \end{array}\right.$$

This step has an intuitive interpretation: the stack is refined by partly deforming each slice towards its neighbors at each iteration. However, in practice we operate with transformations rather than actually transforming the slices, as we discuss in the next section.

#### 2.3.4 Generalization of update neighbors transformations step (replacement of registrations by operations in transformation space)

In the previous section we did not explain how to compute the neighbors transformations $f_{i,i-1}^{m},f_{i,i+1}^{m}$ used in (12). Let's assume that in the first sweep m=1 we have registered each slice to its two neighbors. This gives us neighbor transformations of the form $f_{i,j}^{0},j=i\pm 1$. We then compute the slice updates with (12), obtaining $f_{i}^{0,1}$ and $f_{j}^{0,1}$. Naïvely, we could now transform the images with $f_{i}^{0,1}$ and $f_{j}^{0,1}$ and register them again to compute the neighbors transformations $f_{i,j}^{1}$ from scratch. This is similar to what multi-sweep methods in the literature do, and is a very slow process, because registrations are computationally expensive.

Instead, we can simply update the transformation we had, $f_{i,j}^{1}=f_{j}^{0,1}\circ f_{i,j}^{0}\circ {\left(f_{i}^{0,1}\right)}^{-1}$. That is, we can operate in the space of transformations instead of transforming, interpolating and registering images. As we show in Section 3.2.2, this is several orders of magnitude faster. In the general case, let $f_{i,j}^{m}$ be the solution to the registration from slice *i* to slice *j* at iteration *m*. Let the slice updates computed with (12) be $f_{i}^{m,m+1}$ and $f_{j}^{m,m+1}$. The neighbor transformation can be updated directly in transformation space as

(14) $$f_{i,j}^{m+1}=f_{j}^{m,m+1}\circ f_{i,j}^{m}\circ {\left(f_{i}^{m,m+1}\right)}^{-1}$$

Substituting j=i−1 and j=i+1 in (14) for the two adjacent neighbors, the *update neighbors transformations* expression for each slice becomes

(15) $$\begin{array}{c} f_{i,i-1}^{m+1}=f_{i-1}^{m,m+1}\circ f_{i,i-1}^{m}\circ {\left(f_{i}^{m,m+1}\right)}^{-1},1\leq i\leq N-1\\ f_{i,i+1}^{m+1}=f_{i+1}^{m,m+1}\circ f_{i,i+1}^{m}\circ {\left(f_{i}^{m,m+1}\right)}^{-1},0\leq i\leq N-2 \end{array}$$

Apart from the necessary existence of the inverse, it is desirable that the transformation is closed under inversion and composition. This way, the three transformations in each line of (15) collapse into a single transformation (e.g. the inverse of an affine transformation is an affine transformation, and the composition of three affine transformations is another affine transformation). Otherwise, keeping a list of transformations to apply to each slice becomes cumbersome, and applying them becomes slower as the number of transformations grows. We propose a workaround for this problem in the particular case of B-splines in Section 2.5.

Finally, we note that recomputing registrations may be necessary nonetheless, e.g. if we suspect that they contain errors, typically with larger deformations or artifacts, or a previous registration got stuck in a local minimum. Those cases are due to limitations of the registration algorithm, not the TD formulation, and are beyond the scope of this paper.

#### 2.3.5 Composition of slice transformations

The refinement algorithm proceeds by iterating the slice transformation updates (Section 2.3.3) and neighbors transformations updates (Section 2.3.4). After *M* iterations some stop criterion is met, the refinement algorithm stops, all slice transformation updates are composed, and the total transformation is applied to each slice *I_i_* to obtain the refined slice $I_{i}^{M}$

(16) $$I_{i}^{M}=\left(f_{i}^{M-1,M}\circ \ldots \circ f_{i}^{0,1}\right)\circ I_{i},i=0,\ldots ,N-1,$$

As *M* → ∞, the composition of the transformation updates tends to the inverse of the slice's unknown initial misalignment, $\left(f_{i}^{M-1,M}\circ \ldots \circ f_{i}^{0,1}\right)\to f_{i}^{-1}$. The brackets in (16) emphasize that transformations should be composed and then applied to the slice, to avoid cumulative sampling and interpolation errors.

#### 2.3.6 Stopping criterion

We propose using the number of stack sweeps *M* as the stopping criterion that regulates the amount of smoothing applied by intra-histology refinement. The stopping criterion, in the context of the banana problem (Fig. 3), determines how far away from the noisy banana the refinement moves towards the straight banana. Our model provides a quantitative and theoretical justification. Eq. (4) shows that our diffusion model is equivalent to smoothing the unknown misalignments *f_i_* by a Gaussian kernel. The Full Width at Half Maximum (FWHM) of the kernel is $\text{FWH}M_{Z}=4\sqrt{Dt\;\text{ln}2}$. Substituting the definition of *α* and the time discretization t=MΔt, the spatial width of the kernel can be written in the same units as the slice thickness as ${\text{FWHM}}_{Z}=2\Delta s\sqrt{\alpha M\;\text{ln}2}$. By the properties of the Fourier Transform, the frequency representation of the Gaussian kernel is another Gaussian with bandwidth

(17) $${\text{FWHM}}_{\text{BW}}=\frac{1}{\Delta s}\sqrt{\frac{\text{ln}2}{M\alpha }}.$$

That is, the cut-off spatial frequency for alignment noise is proportional to $\sqrt{1/M}$. In addition, *Δs* is a constant determined by the microtome thickness setting, and *α* can be set a priori as discussed in Appendix A. Thus, empirically estimating a value for *M* also estimates the FWHM that removes alignment noise without overcorrecting, and can be applied to other stacks that have been acquired following a similar protocol. Furthermore, if we change the slice thickness *Δs* or the diffusion step *α*, a value for *M* can be directly recalculated from the FWHM.

To estimate *M* in our experiments, we trace pairs of corresponding landmarks (histology-blockface and histology-histology) and compute the landmark error as the distance between them. As we show in the heart reconstruction experiment of Section 3.2, *M* is not a sensitive parameter, and a wide range of values produces similar reconstruction results.

Alternatively, we considered using the magnitude of the solution update as in Gaffling et al. (2015). But (1) this also requires finding an empirical threshold for the update threshold, similarly to how we find the empirical value of *M*; (2) it has no theoretical support from the model; and (3) it involves defining a meaningful norm for the update of the rigid transformations, combining rotations with translations.

### 2.4 Transformation diffusion reconstruction (TDR)

Combining the update steps above (Section 2.3.3 to 2.3.5), we propose the following Transformation Diffusion Reconstruction (TDR) algorithm (Algorithm 1) based on TD.

Algorithm 1 requires that the transformation is closed under inversion and composition. One of its most important features is that it takes advantage of the following equivalence, derived from Section 2.3.4: *One registration sweep = One sweep of neighbour transformation updates*. Consequently, the computationally expensive process of slice-to-slice registration is only applied at the start (“Registration sweep”), with the loop at step 3) operating only in transformation space, a much faster operation (see Section 3.2.2). This keeps computational times low even when operating on large whole-organ histology datasets. In the following sections we derive expressions for operations ⊙ and ⊕ when applying specific transformations, namely translation and affine.

#### 2.4.1 Translation transformation

Although affine transformations include translations, formulating translation TDR separately leads to vector operations, whereas the affine formulation relies on slower matrix operations. Let a 2D translation transformation be written as

(21) $$f_{\text{trans}}\left(x,y\right)=\left[\begin{array}{c} x\\ y \end{array}\right]+\delta =\left[\begin{array}{c} x\\ y \end{array}\right]+\left[\begin{array}{c} \;\delta_{x}\\ \;\delta_{y} \end{array}\right],$$

where in the notation of Algorithm 1, $f_{i}=\;\delta_{i}$. As with the 1D translation in Section 1, we specify ⊙ and ⊕ are the usual scalar-vector product and vector-vector addition

(22) $$\alpha ⊙\left(f_{i}⊕f_{j}\right)=\alpha \left(\delta_{i}+\delta_{j}\right)$$

The registration sweep requires only N−1 registrations, as $f_{i,j}={\left(f_{j,i}\right)}^{-1}=-f_{j,i}$. Using (22) the update slice transform (18) simplifies for translations to

(23) $$\delta_{i}^{m,m+1}=\left\{ \begin{array}{cc} \alpha \left(\delta_{i,i-1}^{m}+\delta_{i,i+1}^{m}\right), & 1\leq i\leq N-2\\ 2\alpha \delta_{0,1}^{m} & i=0\\ 2\alpha \delta_{N-1,N-2}^{m} & i=N-1 \end{array}\right..$$

Using $\;f_{i}\circ f_{j}=\;\delta_{i}+\delta_{j}$, the update neighbors transformation (19) becomes

(24) $$\begin{array}{c} \delta_{i,i-1}^{m+1}=\delta_{i-1}^{m,m+1}+\delta_{i,i-1}^{m}-\delta_{i}^{m,m+1},\;1\leq i\leq N-1\\ \delta_{i,i+1}^{m+1}=\delta_{i+1}^{m,m+1}+\delta_{i,i+1}^{m}-\delta_{i}^{m,m+1},\;0\leq i\leq N-2 \end{array}$$

and the function composition (16) is computed as

(25) $$\delta_{i}^{m}=\delta_{i}^{m-1,m}+\ldots +\delta_{i}^{0,1},\;i=0,\ldots ,N-1.$$

#### 2.4.2 Affine transformation

Let a 2D affine transformation (of which translation, rigid and similarity transformations are particular cases) be written in matrix form for homogeneous coordinates as

(26) $$\left[\begin{array}{c} f_{\text{aff}}\left(x\right)\\ 1 \end{array}\right]=F\left[\begin{array}{c} x\\ 1 \end{array}\right]=\left[\begin{array}{cc} A & \;\delta \\ 0 & 1 \end{array}\right]\left[\begin{array}{c} x\\ 1 \end{array}\right],$$

where in the notation of Algorithm 1, $f_{i}=\;F_{i}$. There is not a single way to define the operators ⊙ and ⊕, as the linear combination of rotations cannot be optimized simultaneously for torque minimization, constant angular velocity, and commutativity (Bloom et al., 2004). One solution proposed by Alexa (2002), that we use in this paper, is to define linear combinations of affine transformations in their Lie space using the matrix exponential (exp) and matrix logarithm (log)

(27) $$\alpha ⊙\left(F_{i}⊕F_{j}\right)=\exp\left(\alpha (\log F_{i}+\log F_{j})\right),$$

assuming that *F* has a real matrix logarithm. Alexa (2002) proposed that there are no negative eigenvalues if and only if *A* contains no reflections. However, Zacur et al. (2014) have shown that a rotation plus an anisometric scaling in fact produces a transformation with a negative eigenvalue, and thus the matrix logarithm is not real. Nonetheless, Alexa's interpolation is still valid for transformations that are not large, such as the ones in typical histological reconstruction applications such as ours. As with translations, the registration sweep between pairs of neighbors involves only N−1 operations, as the inverse exists for non-degenerate affine transformations, is closed and easily computed, $f_{i,j}={\left(f_{j,i}\right)}^{-1}\Leftrightarrow F_{i,j}={\left(F_{j,i}\right)}^{-1}$. The *Update slice transformation* step (18) becomes

(28) $$F_{i}^{m,m+1}=\left\{ \begin{array}{cc} \exp\left(\alpha \left(\log F_{i,i-1}^{m}+\log F_{i,i+1}^{m}\right)\right), & 1\leq i\leq N-2\\ {\left(F_{0,1}^{m}\right)}^{2\alpha }, & i=0\\ {\left(F_{N-1,N-2}^{m}\right)}^{2\alpha }, & i=N-1 \end{array}\right.,$$

Using matrix multiplication, the *Update neighbours transformations* step (19) becomes

(29) $$\begin{array}{c} F_{i,i-1}^{m+1}=F_{i-1}^{m,m+1}F_{i,i-1}^{m}{\left(F_{i}^{m,m+1}\right)}^{-1},1\leq i\leq N-1\\ F_{i,i+1}^{m+1}=F_{i+1}^{m,m+1}F_{i,i+1}^{m}{\left(F_{i}^{m,m+1}\right)}^{-1},0\leq i\leq N-2 \end{array}$$

and composition (20) becomes

(30) $$F_{i}^{m}=F_{i}^{m-1,m}\ldots F_{i}^{0,1},i=0,\ldots ,N-1$$

### 2.5 Approximated transformation diffusion reconstruction (ATDR)

Histology stacks typically suffer from non-affine transformations. B-splines (Schoenberg, 1946) are one of the most popular non-affine transformations for image registration due to their compact support, sparse representation, availability and speed of computation. In particular, they have been extensively used for histology reconstruction, as noted in the Introduction. However, they cannot be used in Algorithm 1 because: (i) the inverse of a B-spline does not always exist; (ii) when the inverse exists, it is not generally a B-spline and has no explicit parametric form; (iii) composition of two B-splines does not produce a B-spline.

To overcome these obstacles, in this section we provide a modification of TDR, called Approximated TDR (ATDR), which can be applied to B-splines in tensor-product form. The basic idea behind ATDR is to apply TDR to the translations of the control points of the spline. Let a 2D B-spline in tensor-product form (Rueckert et al., 2006) be

(31) $$f_{B-\text{spline}}\left(x,y\right)=\sum_{k=0}^{K}\sum_{l=0}^{L}\left(c_{kl}+\Delta c_{kl}\right)N_{k,p}\left(x\right)N_{l,q}\left(y\right),$$

where $(c_{kl}+\;\Delta c_{kl})\in$ ℝ^2^ are coefficients or control points and *N*_·, *p*_(*x*), the B-spline of order *p*, is a polynomial of order p−1, with $C^{p-1}$ continuity, for example, p=4 for cubic splines. The *c_kl_* components give the coordinates of the (K+1,L+1) points in the control polygon grid, and *Δc_kl_* the translation of the grid points.

For ATDR we propose treating *Δc_kl_* as a translation transformation, and applying the same operators defined above for translations *δ* to vectors $\Delta c={\left[\;\Delta c_{00}\;\Delta c_{01}\ldots \;\;\Delta c_{0L}\;\Delta c_{10}\ldots \;\Delta c_{1L}\ldots \;\;\Delta c_{KL}\right]}^{⊤}$. (We use the boldface notation to differentiate a coefficient *Δc_kl_* from the coefficient vector  *Δc*_*i, j*_ of the B-spline that maps slice *i* onto *j*). That is, we linearly approximate the cubic B-spline by its control polygon. Consequently we approximate the inverse as $f_{B-\text{spline}}^{-1}{|}_{c+\Delta c}\approx f_{B-\text{spline}}{|}_{c-\Delta c}$, and composition as $f_{B-\text{spline}}{|}_{c+\Delta c_{i}}\circ f_{B-\text{spline}}{|}_{c+\Delta c_{j}}\approx f_{B-\text{spline}}{|}_{c+\Delta c_{i}+\Delta c_{j}}$.

This approximation has the advantage that the same TDR apparatus for translations can be reused with three modifications. First, in the registration sweep block, both registrations $f_{i,j}^{m}$ and $f_{j,i}^{m}$ need to be computed for each pair of neighbors, totaling 2(N−1) registrations. Second, the diffusion process for translations requires $\delta_{i,j}=-\delta_{j,i}$ to converge. To achieve this goal, before applying diffusion we adjust the coefficients as

(32) $$\begin{array}{c} \Delta c_{i,j}^{{}^{\prime }}=\frac{\Delta c_{i,j}-\Delta c_{j,i}}{2}\\ \Delta c_{j,i}^{{}^{\prime }}=\frac{-\Delta c_{i,j}+\Delta c_{j,i}}{2} \end{array}$$

And third, as the diffusion of B-spline control points can produce fold-overs in the spline, we use Choi and Lee's (2000) sufficient injectivity conditions (Appendix C). It is worth noting that ATDR will converge to a unique solution that corresponds to maximum alignment of the control polygons, not maximum alignment of the slice images. Nonetheless, our experiments in Section 3.2 suggest that despite this approximation ATDR produces excellent refinement results. Moreover, if one run of ATDR refinement were insufficient for a given data set, the algorithm could be run repeatedly. We summarize these modifications in Algorithm 2.

ATDR features two acceleration factors compared to a naïve baseline algorithm that repeatedly runs registration sweeps. First, replacing registration operations by operations on coefficient vectors is several orders of magnitude faster. Second, in the baseline algorithm each registration sweep produces a new B-spline concatenated to previous transformations. Concatenation of B-splines enables very flexible transformations, but it also makes registration operations increasingly slower as the number of B-spline levels grow (the slowdown is linear in the number of levels). By contrast, ATDR collapses the composition of *M* levels of B-splines into a single B-spline. These two accelerations features come at the price of the approximations described above. In Section 3.2, we show experimentally that ATDR is suitable for histology reconstruction despite those approximations, and in fact can provide more smoothing than the baseline algorithm if required.

## 3 Experiments

### 3.1 Synthetic example for 1D translation diffusion

**Fig. 4:**
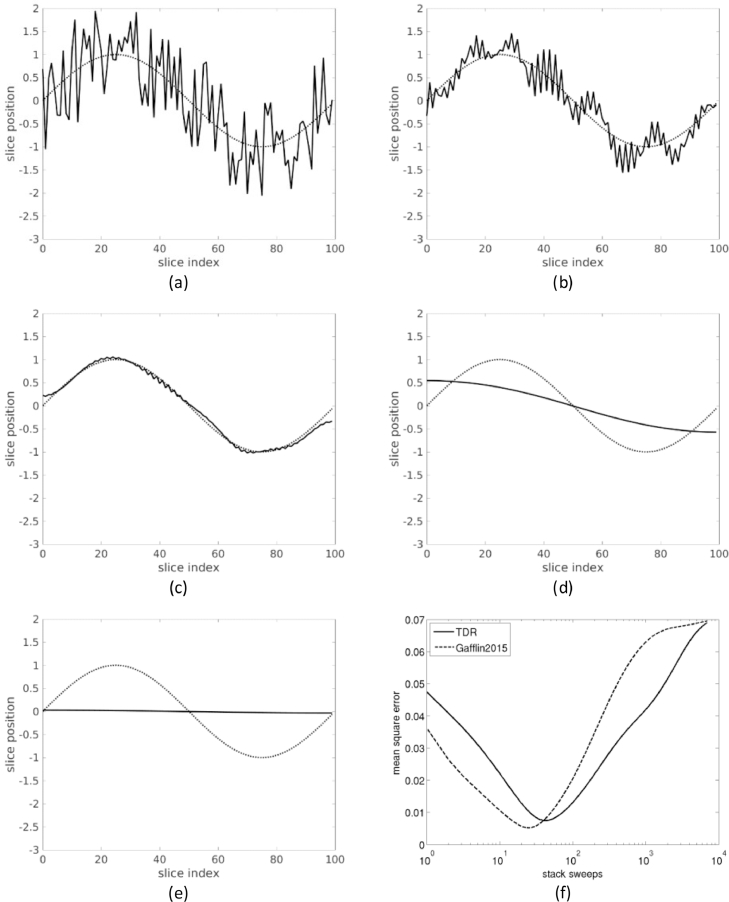
Transformation Diffusion Reconstruction (TDR) applied to a 1D translation synthetic example. First five panels represent the diffusion of a stack of slices (solid line) compared to true shape or ground truth (dotted line). Last panel presents the error between the ground truth and the reconstruction. From top left to bottom right: (a) Initial misaligned stack. (b) After 5 diffusion iterations misalignment is reduced. (c) After 42 diffusion iterations best reconstruction is achieved. (d) After 1000 diffusion iterations the reconstruction has deviated significantly from the true shape. (e) After 7000 diffusion iterations, reconstruction converges to maximum alignment of slices, but far from their true shape. (f) Difference between reconstruction and true shape, measured as mean square error $=∥y_{0}-{\hat{y}}_{0},\ldots ,y_{N-1}-{\hat{y}}_{N-1}∥/N$ for 7000 stack sweeps of our TDR method (solid line) and Gaffling et al.'s (2015) Gauss Seidel approach (dashed line). The minima corresponding to the best reconstructions are obtained after 42 (TDR) and 25 (Gaffling) diffusion iterations, and are $7.42\cdot 10^{-3}$ and $5.16\cdot 10^{-3}$, respectively. The best reconstruction error worsens by 10% after 14 (TDR) and 6 (Gaffling) stack sweeps.

To illustrate the method, we use a synthetic example for 1D translation, illustrated in Fig. 4. First, the vertical position *y* of 100 slices was generated as $y_{i}=\sin\left(2\pi i/N\right)+2.4ɛ$, where ɛ is random noise uniformly distributed in [−0.5,0.5], simulating registration noise from histology to an external reference like blockface (Fig. 4a), i.e. the noisy banana. The sinusoidal component ${\hat{y}}_{i}=\sin\left(2\pi i/N\right)$ is the ground truth (true shape) that we ideally want to reconstruct. For simplicity, we then assumed that intra-histology registration is perfect, and computed $f_{i,j}^{0}=y_{j}-y_{i}$, without a noise term. (In a real problem, we would not know any *y_i_*, and the initial transforms $f_{i,j}^{0}$ would therefore be the result of registering each slice to its neighbors). Then, we applied only diffusion operations in transformation space to reconstruct the stack. After 5 iterations (Fig. 4b), the registration partly reduced the registration noise. After 42 iterations (Fig. 4c), the reconstruction method achieved the closest result to the true shape (curved banana). After 1000 iterations (Fig. 4d), all registration noise was removed, but the reconstruction had substantially diverged from the true shape. After 7000 iterations (Fig. 4e), the slices converged to maximum alignment, and all information about the true shape was lost (straight banana). The evolution of the error between the true shape and the reconstructed stack is shown in Fig. 4f. The error initially decreases, as registration noise is removed, here with an optimum at 42 iterations, and then increases, as the information about the true shape of the stack starts to be lost. In Fig. 4f we also show the error from Gaffling et al.'s (2015) Gauss Seidel back and forth approach applied to the same noisy example. After 7000 stack sweeps, Gaffling's approach has converged to roughly the same maximum alignment solution as TDR. Gaffling's method reaches optimal reconstruction sooner, after 25 stack sweeps. This is to be expected, as sequential methods apply the smoothing per slice rather than per stack sweep, so they effectively accumulate more smoothing. Conversely, we would expect that they degrade the true shape solution in fewer sweeps too. As shown in Fig. 4f, the reconstruction error worsens by 10% after 14 extra sweeps in TDR, and only 6 in Gaffling's method. Thus, stopping criteria for the latter would require finer tuning. Gafflin's method achieves a slightly better optimum, though, with minimum square error (MSE) $5.16\cdot 10^{-3}$ vs. $7.42\cdot 10^{-3}$ for TDR.

**Fig. 5:**
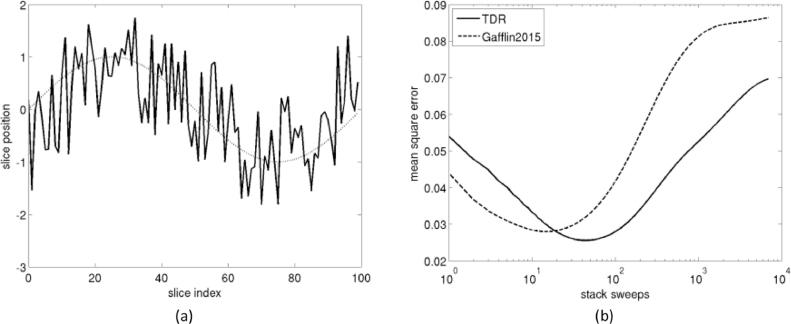
Previous synthetic example with small drift component added to the random error. (a) Initial misaligned stack (solid line) and true shape ground truth (dotted line). (b) Difference between reconstruction and true shape, measured as mean square error $=∥y_{0}-{\hat{y}}_{0},\ldots ,y_{N-1}-{\hat{y}}_{N-1}∥/N$ for 7000 stack sweeps of our TDR method (solid line) and Gaffling et al.'s (2015) Gauss Seidel approach (dashed line). The minima corresponding to the best reconstructions are obtained after 44 (TDR) and 15 (Gaffling) diffusion iterations, and are $2.56\cdot 10^{-2}$ and $2.80\cdot 10^{-2}$, respectively. The best reconstruction error worsens by 10% after 63 (TDR) and 24 (Gaffling) stack sweeps.

For the second synthetic experiment, we add a small drift component –i.e. correlated error– to the random error, $y_{i}=\sin\left(2\pi i/N\right)+2.4ɛ+10^{-2}i-0.5$ (Fig. 5a). As expected, drift degrades Gaffling's sequential method more than TDR, which is parallel (Fig. 5b). The minimum MSE values are similar, $2.56\cdot 10^{-2}$ (TDR) and $2.80\cdot 10^{-2}$ (Gaffling). TDR is again less sensitive to the tuning of stop criterion parameters, as the error degrades 10% after 63 extra sweeps vs. 24 extra sweeps for Gaffling's method.

These synthetic examples are useful to illustrate the banana problem of Section 2.3.1. In particular, how refinement sweeps shift the solution from noisy banana to true shape to straight banana. The optimal number of iterations *M* can be empirically determined by measuring landmark distances between the reconstruction and an external reference or ground truth. As shown in Section 2.3.6, *M* determines the bandwith of the smoothing kernel. Thus, an optimal *M* can be estimated in one stack and then applied to similar data. In the synthetic examples above (that present pure frequency components), overshooting the optimal *M* by 33% (no drift component experiment) to 143% (drift component experiment) degrades the solution only by 10%, which suggests that the solution is not very sensitive to the choice of *M* (note that the *M* axis is logarithmic). In the next section, reconstruction of real data also shows that a wide range of values of *M* produce similar reconstruction results.

### 3.2 Heart reconstruction

We performed reconstruction experiments on the stack of Picro Sirius Red slices (10 µm thickness, spaced 20 µm) covering a mouse heart (Siedlecka et al. (2017), see Section 2.1 for wet lab details), after removing severely damaged ones for a total of 239 slices. Initial results of these experiments for half the heart were reported in Casero et al. (2016). The histology reconstructed in this section can be downloaded from Casero et al. (2017).

As it is common in registration problems, we applied a two-level reconstruction approach (low resolution followed by high resolution), with the preprocessing and registration algorithms detailed in Section 2.2. **Low resolution reconstruction:** Histology slices were downsampled by a factor of × 24.6 down to blockface pixel size, and pre-aligned to the blockface using rigid registration, followed by rigid TDR refinement and B-spline ATDR refinement. **High resolution reconstruction:** The low resolution reconstruction was applied to high resolution histology downsampled by a factor of × 2, followed by high resolution B-spline ATDR refinement. This downsampling still shows the outline of individual cells, and thus contains all microstructure information, but reduces the registration sweep time by a factor of 4. We set α=0.45 (Appendix A) and studied reconstruction results for a range of diffusion sweeps *m*.

Validation of histology reconstruction is challenging. Similarity measures are misleading for registration validation, and distance errors should be used instead (Rohlfing, 2012). Blockface images have poorer resolution than histology even when the latter is downsampled to the same pixel size (Fig. 7) —other external references have limitations too, as discussed in the Introduction. In practice, for blockface it is possible to hand trace landmark correspondences that are adequate for low resolution validation, but there is not enough detail in the images for high resolution refinement. Thus, for low resolution reconstruction we use both landmark errors and qualitative visual assessment, and for high resolution refinement, only qualitative visual assessment.

We compute two adversarial types of landmark errors for quantitative validation, (1) blockface-histology to quantify the deviation from the true shape and (2) histology-histology between pairs of adjacent slices to quantify stack smoothing. They are adversarial according to the maximum alignment discussion in Section 2.3.1, as optimization of blockface-histology errors produces the noisy banana solution, whereas optimization of histology-histology errors tends to the straight banana solution. We calculate the median and 95th percentile landmark error curves, as they reflect the effect of refinement on typical and larger landmark errors, while avoiding outliers.

For quantitative evaluation we created a set of 411 pairs of blockface-histology landmarks (7 to 23 pairs of landmarks per slice in 28 slices covering the stack) and a set of 1494 pairs of intra-histology landmarks (8 to 76 pairs of landmarks per pair of adjacent slices for 40 pairs). Landmarks were primarily placed in the center of the cross-section of small vessels, edges of larger vessels, and the pointed cusps and valleys of trabeculae and cleavage planes. Blockface-histology landmark errors were computed as ${ɛ}_{ij}=∥p_{ij}-q_{ij}∥$ , where *p_ij_, q_ij_* are the *j*-th landmark in the *i*-th histology and blockface slices, respectively. Histology-histology errors were computed as ${ɛ}_{ij}=∥p_{ij}-p_{i+1,j}∥,$.

#### 3.2.1 Low resolution reconstruction without external blockface reference

**Fig. 6:**
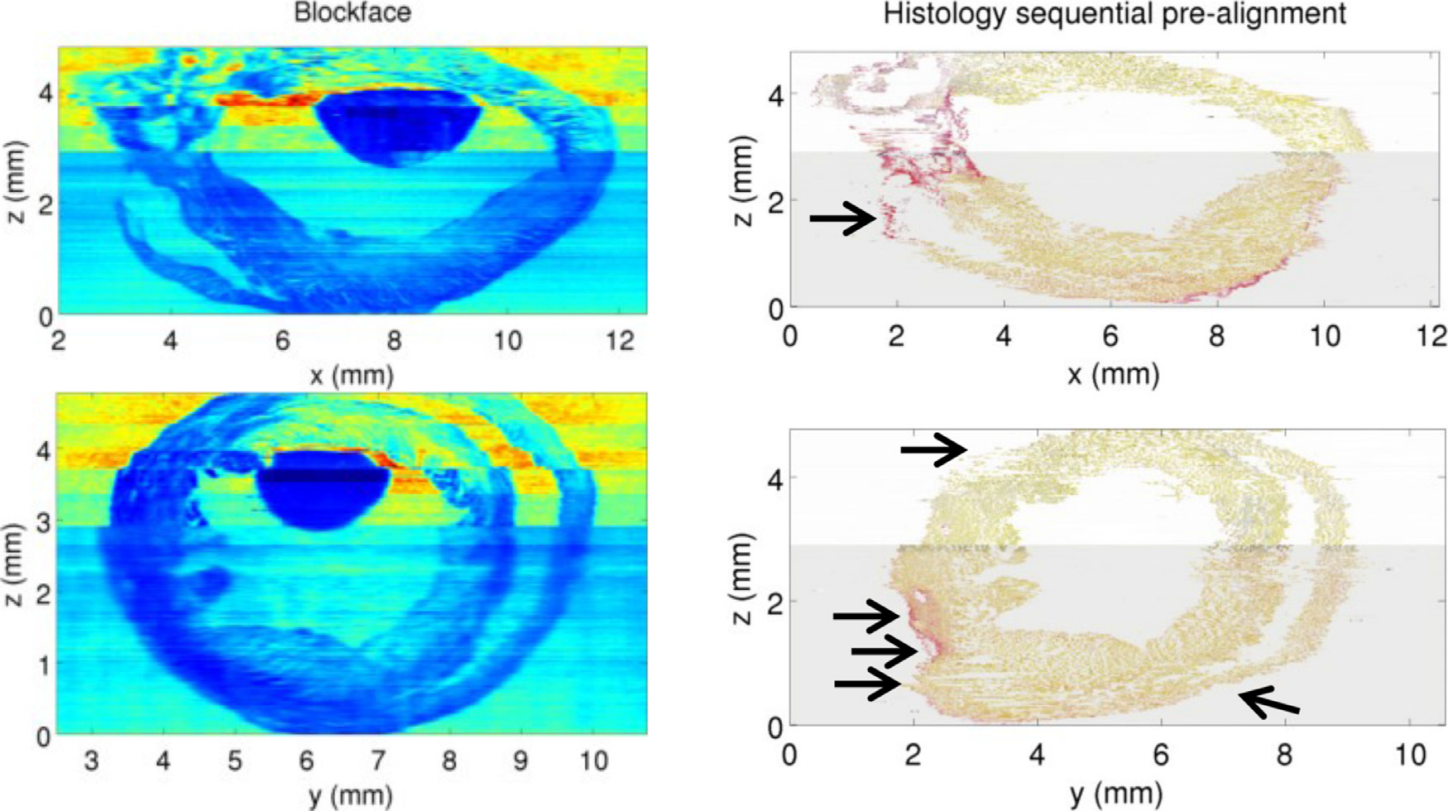
Sequential rigid pre-alignment of histology without blockface (right). Alignment starts from central slice and propagates towards the top and bottom slices, separately. Virtual slices show middle long axis (XZ plane) and short axis (YZ plane) cuts. Corresponding blockface virtual slices (left) are provided as visual reference, but were not used for the reconstruction. Black arrows point to drift artifacts. In addition, the short axis of the histology is skewed. The discontinuity in the blockface virtual slices near Z = 3 mm is caused by a few missing slices, and the dark blob corresponds to a bubble in the wax block.

Yushkevich et al. (2006) showed in mouse brain that sequential registration of histology slices without an external reference produces a smooth reconstruction but suffers from large-scale geometric artifacts caused by drift. They qualitatively compared histology virtual slices to an MRI external reference. Casero et al. (2016) showed a similar quantitative result for mouse heart using landmark distance errors. This section complements (Casero et al., 2016) qualitatively, showing virtual slices of histology compared to virtual slices of the blockface external reference (Fig. 6). For this first experiment, we started the sequential alignment from a central histology slice, *I*_121_, and propagated it towards the top and bottom slices, by rigid registration of *I_i_* to $I_{i+1}$, i=120,…,1, and *I_i_* to $I_{i-1}$, i=122,…,239. (Starting from the middle reduces the amount of accumulated error compared to starting from an end slice.) Drift artifacts appear in the short axis virtual slice as wobbles in the cardiac wall and a skewed cardiac shape. Refinement (or regularization) of this pre-alignment would smooth the reconstruction, but cannot remove drift artifacts and recreate the missing true shape information, as discussed in Section 2.3.1, by Yushkevich et al. (2006) and shown experimentally in Casero et al. (2016).

#### 3.2.2 Low resolution reconstruction with external blockface reference

In the second experiment, we performed a blockface-aided reconstruction replacing the previous sequential histology-histology pre-alignment by a rigid downsampled histology-blockface pre-alignment. This was followed by intra-histology refinement in two stages: low resolution rigid TDR and low resolution B-spline ATDR.

**Fig. 7:**
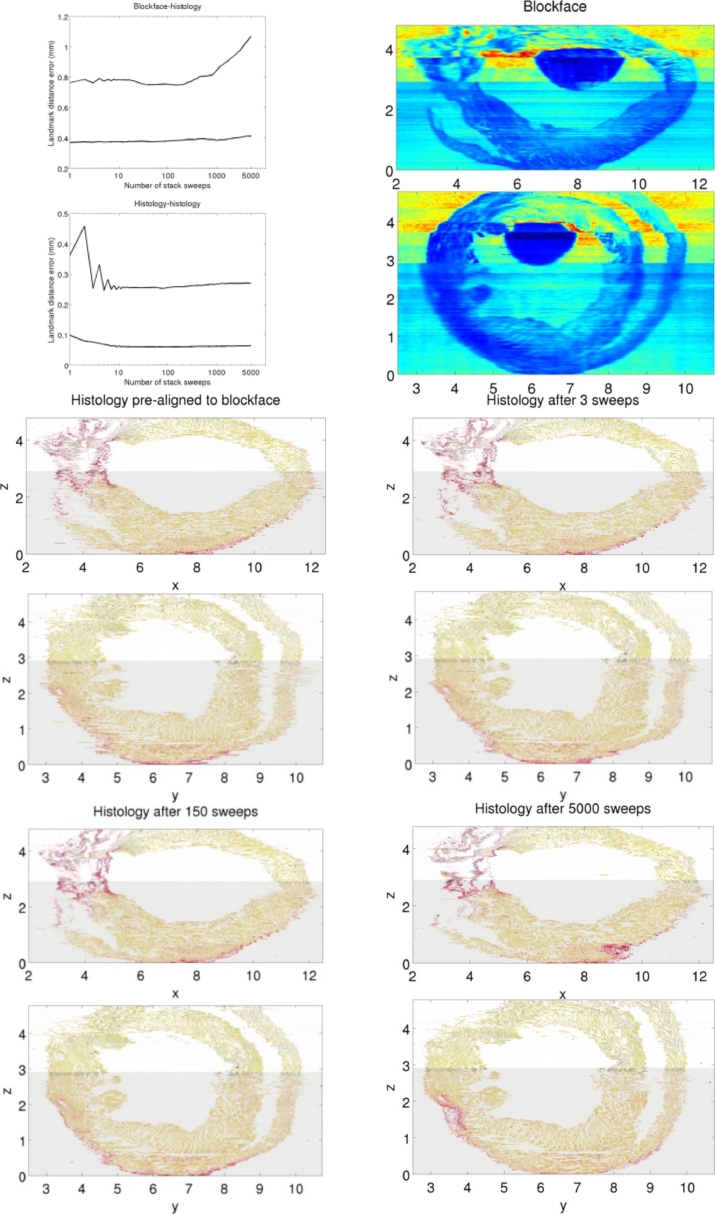
Rigid intra-histology refinement of mouse heart after histology-blockface pre-alignment. Blockface-histology and histology-histology landmark error for rigid refinement (median and 95th percentile) followed by long axis (XZ plane) and short axis (YZ plane) virtual slices to illustrate different number of stack sweeps. Blockface virtual slices provided as reference for anatomical true shape. Landmark error figures use a logarithmic scale for number of stack sweeps. Virtual slices’ axes in mm. Results between m=10 and m=400 are quantitatively and qualitatively similar to the virtual slices displayed for m=150. Under and overcorrection are illustrated with m=3 and m=5000, respectively.

Low resolution rigid TDR refinement is analyzed in Fig. 7. For the blockface-histology error, the median curve gently increases with the number of sweeps *m*, whereas the 95th percentile curve has a global minimum at m=150 sweeps. At the same time, median histology-histology error decreases smoothly with the number of stack sweeps. The 95th curve initially oscillates, as diffusion smooths out larger misalignments. Visual inspection of the virtual slices in Fig. 7 suggests that M=150 produces a satisfactory result. To illustrate under and overcorrection we also display virtual slices for m=3 (a local minimum) and m=5000 (showing vertical straightening of the right ventricle). As our method replaces registration operations by affine matrix operations form *m* > 1, the computational cost of refinement with M=150 sweeps is negligible. This also allows producing virtual slices for several values of *m* for visual inspection. Both plots combined show a large range (m=10 to m=400) with small quantitative differences, suggesting that the final result is not sensitive to the choice of stopping criterion *M* within a wide range of values. Overcorrection is more difficult than undercorrection, as transformation updates become smaller with the number of sweeps (note that the *m* axis is logarithmic).

**Fig. 8:**
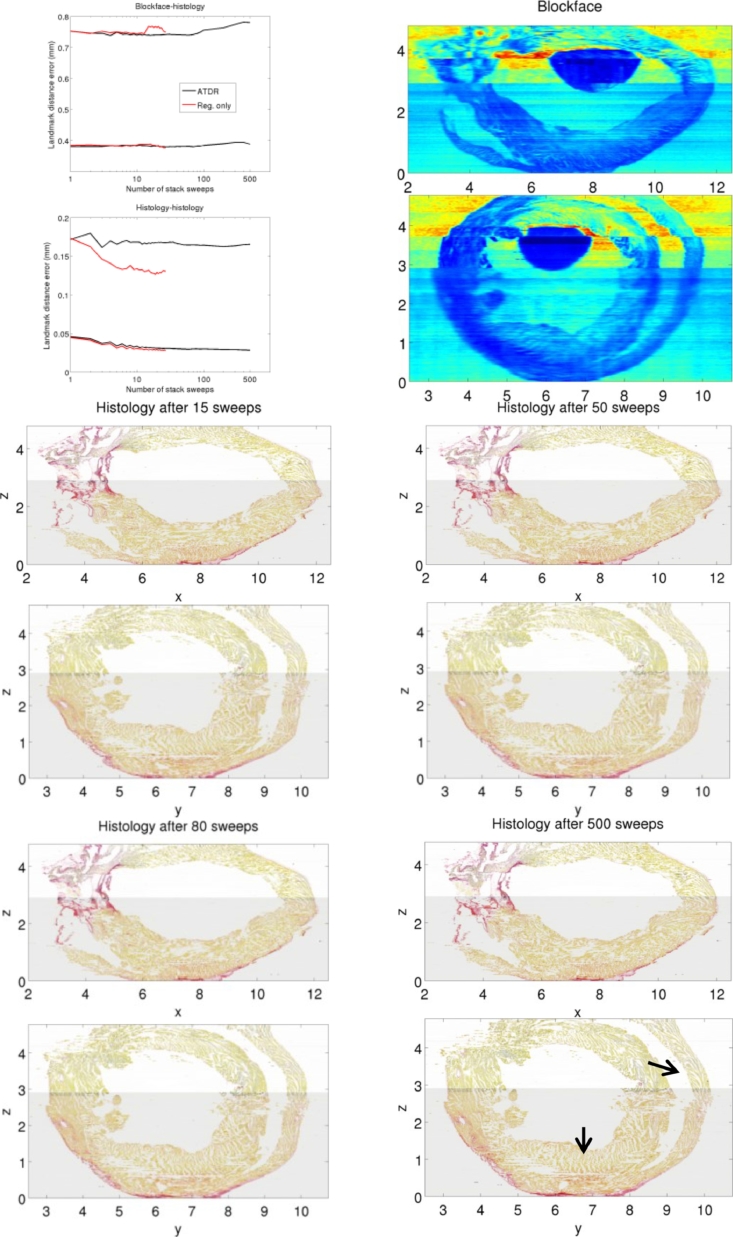
Low resolution B-spline intra-histology refinement of mouse heart after rigid TDR intra-histology refinement with M=150 stack diffusion sweeps. Comparison of a baseline approach where stack sweeps use registrations only (red error curve) vs. our ATDR approach that uses a single B-spline level and replaces registrations by diffusion operations in transformation space (black error curve). Blockface-histology and histology-histology landmark error (median and 95th percentile) followed by long axis (XZ plane) and short axis (YZ plane) virtual slices to illustrate different number of stack diffusion sweeps *m*. Landmark error figures use a logarithmic scale for *m*. Blockface virtual slices provided as reference for anatomical true shape. Virtual slices’ axes in mm. Results between m=15 and m=80 are quantitatively and qualitatively similar. Overcorrection is illustrated with m=500, both in the straightening of the Right Ventricle's bend and the verticalization of cleavage planes in the Left Ventricle's inferior wall (black arrows). (For interpretation of the references to colour in this figure legend, the reader is referred to the web version of this article.)

Low resolution B-spline refinement, applied after the rigid refinement, is shown in Fig. 8, comparing ATDR to a naïve baseline smoothing algorithm that only applies registration sweeps. We ran the baseline algorithm only up to m=27 registration sweeps, as beyond that level the concatenation of B-splines made the registration sweeps too slow. We use the results in Fig. 8 to assess whether ATDR produces an adequate refinement. The median and 95th percentile curves for blockface-histology error are similar for ATDR (black) and the baseline algorithm (red), suggesting that both approaches similarly preserve the true shape of the specimen. The histology-histology curves and visual results have negligible change between m=10 and m=80. Thus, the refinement is not very sensitive to the choice of stopping criterion *M* within that range. The curves also suggest better smoothing by the baseline algorithm, as expected. However, after the first registration sweep, ATDR is five to seven orders of magnitude faster than the baseline algorithm (times provided below). Thus, in practice, it is possible to run more sweeps with ATDR in a fraction of the time, and without being limited by the number of B-spline levels. The 95th percentile curve, on the other hand, is significantly better for the baseline algorithm. However, visual inspection of virtual slices does not show noticeable differences. Moreover, virtual slices in Fig. 8 not only show that ATDR smooths adequately, but it can even overcorrect, e.g. with m=500 if required for testing.

We now provide run time and memory requirements for low resolution refinement. A rigid registration sweep for the downsampled histology stack with 239 slices required 238 registrations, taking ∼ 1.6 · 10^4^ s on a workstation with 8 Intel Xeon 3.70 GHz cores. (Note that this is very slow, ∼4.5 h, but we aimed to have a very robust rigid method that we can measure improvements against. TDR itself is independent of the speed and other implementation details of the registration algorithm). The equivalent neighbor transformation update from TDR (Algorithm 1) operated with (3, 3)-matrices. Each of the 238 updates took ∼ 1.0 s to compute 2 matrix logarithms, 1 matrix exponential, 5 matrix multiplications and 2 matrix inversions. A B-spline registration sweep for the low resolution stack involved 476 registrations, taking ∼ 9.8 · 10^2^ s for m=1 and increasing linearly with *m* to ∼ 2.5 · 10^5^ s for m=27. The equivalent neighbor transformation update operated with 238 vectors, each of length 1150. Each update in transformation space took $∼4.5\cdot 10^{-3}$ s to compute 11 vector additions and 5 multiplications by a scalar. That is, neighbor transformation updates were 4 (rigid) to 5 or 7 (B-spline) orders of magnitude faster than equivalent registration sweeps. In terms of memory use, each registration to two neighbors stored three 971 × 1099 pixels images, plus two similar transformed images, using around 5.3 M. An equivalent neighbor transformation update stored 4 (3, 3)-matrices, using 288 bytes (rigid), or 4 vectors of length 1150, using 31 K (B-spline). That is, neighbor transformation updates used between 2 (B-spline) and 4 (rigid) orders of magnitude less memory.

#### 3.2.3 High resolution refinement

For the third experiment, we applied the low resolution transformations computed above to the original histology downsampled only by a factor of 2. These are very high resolution images that show microstructure detail of individual cell shape and cleavage planes. After applying the low resolution transformations, we obtained a stack of 239 RGB slices with size 9341 × 12,552 pixels that would take a total of 84G in memory. Preprocessing of the histology converts the three RGB channels to one, and the size reduces to 28G. This volume of data made it necessary to run our algorithms loading and saving individual slices from the hard drive as required, rather than loading the whole stack in memory, as it is possible for low resolution reconstruction.

**Fig. 9:**
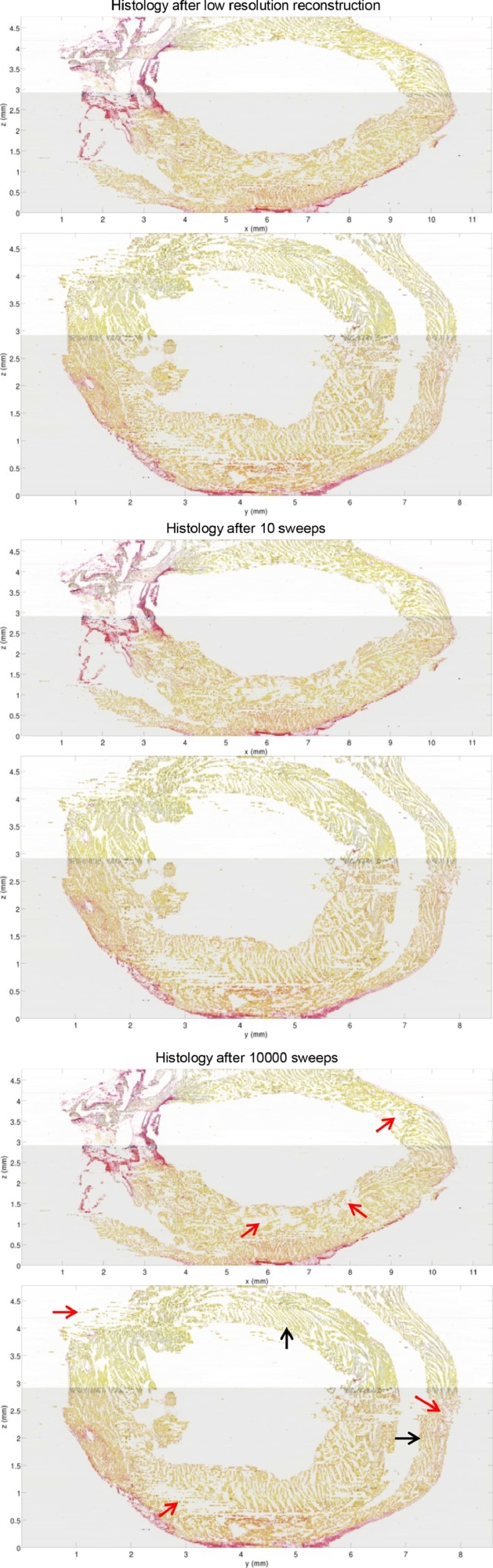
High resolution B-spline ATDS intra-histology refinement of mouse heart after low resolution reconstruction with M=150 rigid TDR stack diffusion sweeps and M=80 B-spline ATDR sweeps. Virtual slices show middle long axis (XZ plane) and short axis (YZ plane) cuts for several number of diffusion sweeps *m*. Results between m=5 and m=100 are qualitatively similar. Undercorrection is illustrated with application of low resolution reconstruction without any further refinement (equivalent to m=0). Correct refinement is illustrated with m=10. Slight overcorrection is illustrated with m=10,000, both by a small shift of the Right Ventricle's endocardium and angle change of cleavage planes in the septum (black arrows). We also point to some areas where tissue damage caused by Karnovsky's fixation did not allow reconstruction of the microstructure (red arrows). (For interpretation of the references to colour in this figure legend, the reader is referred to the web version of this article.)

Landmark validation is not feasible at high resolution, as discussed above. For visual qualitative evaluation we refined the histology with m= 1, 3, 5, 7, 10, 20, 30, 40, 50, 100, 500, 1000, and 10,000 ATDS diffusion sweeps in transformation space after the registration sweep. After the initial 5.8 h registration sweep, at ∼ 10 s per diffusion sweep, producing refined stacks for testing took another 10 s (1 diffusion sweep) to 1 day 3 h (10,000 diffusion sweeps). Note that after the registration sweep, B-splines for the whole stack can be stored and operated with in computer memory, avoiding slow file input/output operations (sizes provided below). The results are shown in Fig. 9. We produced virtual slices for the central long axis and short axis of each stack, by loading one slice at a time and keeping in memory only the row and column that belong in the virtual slices. Producing virtual slices took ∼15 min per stack. Changes in the microstructure were subtle enough, but they were revealed by scrolling back and forth through the virtual slices. For m=1 and m=3 diffusions the microstructure was undercorrected and spatial noise was apparent. Between m=5 to m=100, slice-to-slice transitions in the microstructure looked smooth and changes were very subtle, which gives a wide range of valid values for the stopping criterion *M*. Above m=500, changes in cleavage plane angles and displacement of endocardial and epicardial walls were small but noticeable.

High resolution refinement of the 239 slices used 476 B-splines. Each B-spline is parametrized by 195,480 coefficients, taking 1.6 M in memory. By comparison, a preprocessed histology image takes 117 M in memory. The B-spline registration sweep took ∼ 2.1 · 10^4^ s, whereas equivalent neighbor transformation updates took ∼ 10 s per sweep. Thus, B-spline sweeps in transformation space require 73 times less memory and are 3 orders of magnitude faster than the fastest registration sweep (1st level of B-splines).

Visual assessment of the virtual slices for stopping criterion M=10 shows smooth slice-to-slice transitions in the image, showing microstructure such as cleavage planes in great detail. Cleavage planes meeting at sharp angles and the pointy tip of the Left Ventricle suggest that, although smoothing has removed high frequency registration noise, it has not necessarily removed high frequency anatomical features. A verticalization distortion is noticeable at the top slice of the Right Ventricle's epicardium. As the very top slices of the stack are missing due to damaged tissue, we hypothesize that the Neumann boundary condition has promoted the epicardial wall to be parallel to the Z-axis in the top slice, although this artifact is not apparent in the rest of the top slice or in the bottom slice. The virtual slices also show a few areas where the microstructure is not reconstructed (red arrows in Fig. 9). These are artifacts caused by tissue tears due to the tissue becoming brittle from the use of Karnovsky's fixative for work unrelated to this paper, that do not occur in this form in regular histology processing. Tissue tears represent a transformation discontinuity that cannot be corrected with the usual smooth transformations, and are beyond the scope of this work.

## 4 Discussion

We have proposed a new approach that produces smooth and anatomically sound high-resolution 3D reconstructions of tissue from serial 2D histology data without compromising overall shape, thanks to the combination of a blockface reference and a mathematical framework called Transformation Diffusion (TD). We present the reconstruction of a full mouse heart, 239 RGB slices with slice size 9341 × 12,552 pixels, pixel size 0.92 µm × 0.92 µm, cut 10 µm thick, spaced 20 µm (84G). Our approach is independent of the registration methods used, as it only depends on the properties of the resulting transformations.

The TD framework leads to simple and intuitive refinement algorithms that are similar to previous radius-1 heuristics, that we call Transformation Diffusion Reconstruction (TDR) for translation/affine transformations and Approximated TDR (ATDR) for tensor-product B-spline transformations with guaranteed injectivity. They are trivially parallelizable, have no bias as they have no starting slice, and by contrast to sequential methods, errors do not accumulate along stack sweeps. We have shown a mathematically sound way of replacing registration operations by operations in transformation space that are several orders of magnitude faster and less memory-demanding. Thus, our uncoupled parallel algorithms in practice run as fast as a sequential causal algorithm. We have shown that our radius-1 algorithms produce smoothing that is equivalent to larger neighborhoods. We have also provided theoretical and numerical analysis for the algorithms’ two parameters. The diffusion step *α* determines the implicit smoothing kernel. To reduce the number of stack sweeps, *α* can be set to the largest value that avoids high-frequency noise (we show α=0.45 to be adequate). However, because stack sweeps in transformation space are so fast, setting lower values for *α* would have a small performance impact. We have shown that TD is equivalent to Gaussian smoothing of the deformations where the number of stack sweeps *M* determines the Gaussian bandwidth as $\text{FWH}M_{\text{BW}}\propto 1/\sqrt{\alpha M\;}$ for translations, and FWHM_BW_ has the same units as the inverse of the slice thickness. Our experiments suggest that *M* provides a non-sensitive stopping criterion that can be estimated empirically using landmark errors and visual assessment for translations, affine and B-spline transformations. Future work will complement these empirical results with a theoretical study of the meaning of this Gaussian filtering in the Lie space of affine transformations and B-spline control point translations.

ATDR approximates cubic B-splines by their control polygon, collapse several levels of B-splines into one, and constraint control point displacement to ensure injectivity. Despite these approximations, ATDR produces similar refinement quality as multiple B-spline registration sweeps, as we show in our heart reconstruction experiments. Future work will study extending TDR to other types of non-affine transformations. Interesting candidates are linear B-splines, as their control polygon coincides with the spline, and displacement and velocity fields (Vercauteren et al., 2007), as the vectors could be diffused with translation TDR.

The TD framework provides insight into the classical banana problem as a spectrum of solutions between the noisy stack and “maximum alignment” or the straight banana solution. Namely, the Gaussian filter equivalence provides an intuitive explanation to why the method removes high frequency registration noise before smoothing lower frequency anatomical features.

We use Neumann boundary conditions as Gaffling et al. (2015) for the top and bottom slices of the stack. They allow free movement of those slices, but they would tend to make the tangent of the reconstruction along the Z-axis horizontal. This does not seem to affect the heart reconstruction in general, except seemingly at the top slice of the right ventricle's epicardium, and it is slightly present in the synthetic experiments. Thus, alternative boundary conditions are another interesting topic for further research.

Let a neighborhood with a linear combinations of transformations be $\phi_{i}^{m,m+1}=\sum_{k=-d}^{d}\gamma_{k}\phi_{i+k}^{m}$. Our slice update in (10) can be seen as a linear combination of transformations with neighborhood $\gamma_{\pm 1}=\alpha$. As advanced in the Introduction, some authors have proposed using larger neighborhoods. Linear combination neighborhoods use e.g. binomially distributed weights $\gamma_{k}=\left(\begin{array}{c} 2d\\ k+d \end{array}\right)/2^{2d}$ (Ju et al., 2006) or Gaussian weights $\gamma_{k}=\text{exp}\left(-k^{2}/4\right)$ (Rusu et al., 2015). These larger neighborhoods produce more smoothing, but in Appendix B we show that *d* radius-1 sweeps of (10) produce the same smoothing as one radius-*d* sweep. The radius-*d* neighborhood requires d(N−1) per stack sweep, though, whereas *d* stack sweeps with our radius-1 neighborhood in practice cost the same as (N−1) registrations. Hence, TDR is *d* times faster than if we used a radius- *d* neighborhood. This is significant, as our heart experiments require typical values of 10 ≤ *d* ≤ 150 (Section 3.2).

A study of other neighborhood characteristics is beyond the scope of this work. However, we provide some observations for future work. Larger neighborhoods may seem more robust against registration errors. However, the further two slices are located from each other, the less similar they look, and the more likely it is for the registration to be meaningless or fail. For example, a large neighborhood could try to register two sets of cleavage planes at different angles, trabeculae with different topology, the cross-section of an artery to the two branches it bifurcates into, or an atrial slice to a slice where only part of the right ventricle is visible. Thus, they can introduce a systematic source of registration noise and artifacts.

TDR and ATDR can be applied equally to reconstructions initialized with or without an external reference such as blockface. But the results from our experiment in Section 3.2.1, in agreement with previous reports (Streicher et al., 2000, Yushkevich et al., 2006), strongly suggest that reconstruction methods that omit an external reference are prone to produce large geometric artifacts. Moreover, the validation of those methods may be misguided by intra-histology error measures, as it is possible to reduce the slice-to-slice error without reducing large geometric artifacts. This problem may not affect all tissue samples equally. For example, small rectangular blocks with symmetric microstructure may be more resilient to reconstruction artifacts. For larger samples like ours, as well as samples with asymmetric structures that are not normal to the cutting plane, large errors should be expected.

In the synthetic experiments of Section 3.1 we compared our TDR approach to the Gauss–Seidel method of Gaffling et al. (2015). The results suggest that our approach is less sensitive to the choice of the stopping parameter, and that Gauss–Seidel achieves a slightly better reconstruction if there is no correlated noise, and TDR if there is. In practice, our approach is much faster, because of the replacement of registrations by operations in transformation space, although future work could study whether a similar trick could be applied to Gaffling's method. Gaffling's registration of *I_i_* to $I_{i-1}\circ \frac{1}{2}\phi_{i-1,i+1}$ also introduces an additional source of error that we did not take into account in our synthetic experiments, and could worsen their results in real data.

In the mouse heart experiments (Section 3.2), our brute-force matched filter rigid registration approach is relatively slow (∼67.2 s per slice compared to 2.1 s for B-spline registration of the low resolution histology), but extremely robust, as it provides a global optimum and is optimal in a signal-to-noise ratio (SNR) sense. Indeed, all histology slices obtained a good initial alignment to the blockface, despite the presence of tissue tears, fold-overs, non-affine deformations, and bubble artifacts in the histology images, and smears, scratches, or reduced contrast in the blockface images. The matched filter rigid registration method has a simple Matlab implementation, whereas the B-spline method benefits from many ITK features that improve speed, such as the ITK C+++ multi-thread implementation, gradient descent, multi-level registration, binary masks to ignore most of the image, or subsampling of the error metric. Future work will look into adapting these and other speed improvements such as phase correlation with fast rotation estimation (Reddy and Chatterji, 1996) without losing robustness.

While this paper provides interesting theoretical and implementation ideas, there are several directions for further development and application to histology reconstructions. For instance, we have followed the assumption from the literature that anatomical structures vary slowly with respect to slice thickness (Guest and Baldock, 1995, Ju et al., 2006, Saalfeld et al., 2012, Wirtz et al., 2004), but it is debatable whether this is true at local histological discontinuities, such as vessel bifurcations or starting points of structurally distinct features (trabeculae, papillary muscles, septum-free wall intersection). The results from our high resolution experiment (Section 3.2.3) suggest so, but due to the lack of a true gold standard, we may be smoothing out some microstructure detail without noticing. This would open an interesting line of future work, where treating the diffusion coefficient *D* as a tensor, using Fourier's diffusion equation (Narasimhan, 1999) instead of the heat equation could lead to novel anisotropic diffusion schemes. Furthermore, analysis of the characteristic of registration noise and sample structures frequencies could find an optimal number of stack sweeps *M* without having to analyze landmark errors. We discussed in Section 2.4.2 that Alexa's (2002) affine interpolation scheme is known to fail in some cases, so studying alternatives could improve the range of application of the algorithms. Finally, applying our algorithms to tissue with microstructure that differs from cardiac (such as brain or lung) would be an attractive endeavor.

## Figures and Tables

**Fig. 10:**
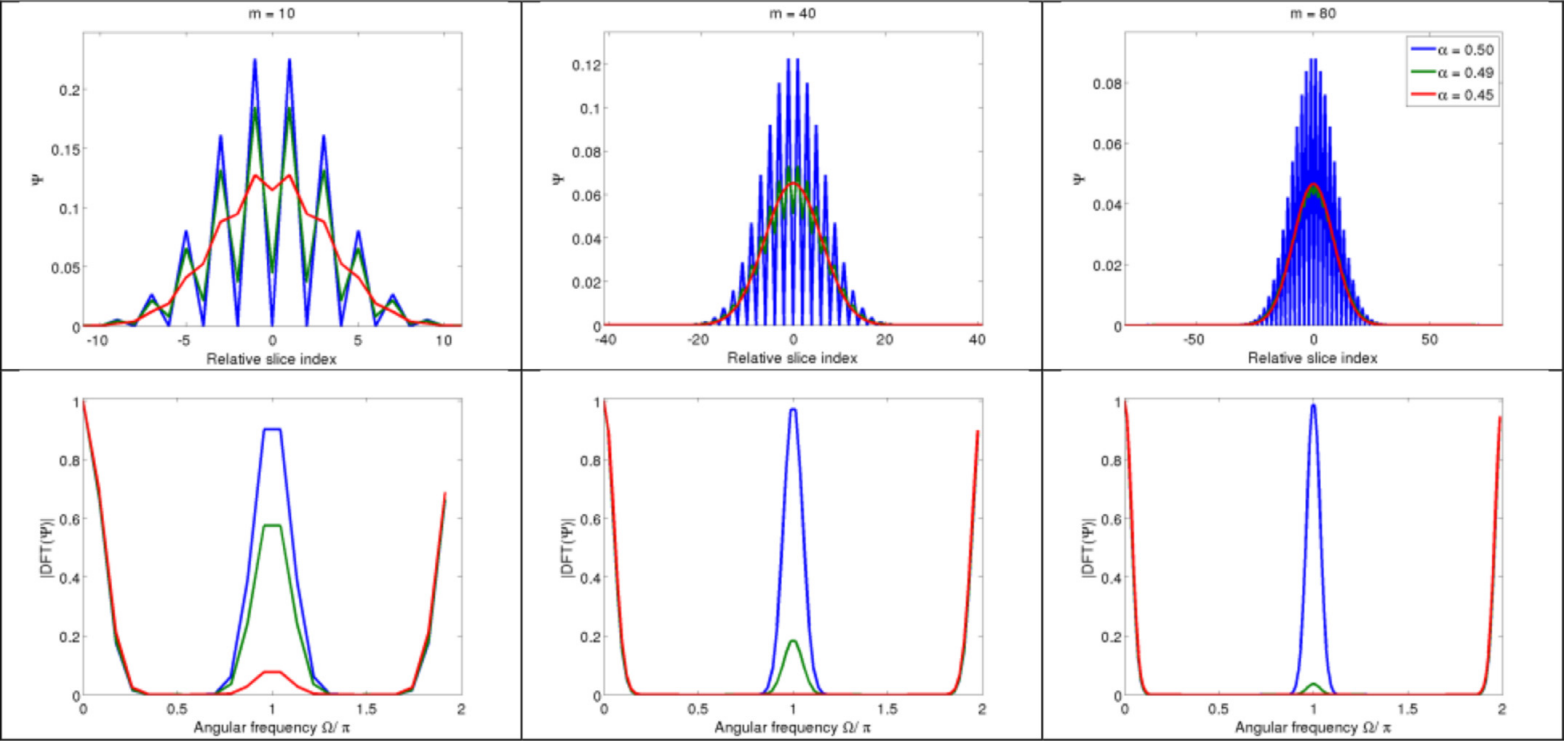
TDR/ATDR smoothing kernel *Ψ* for several number of repeated convolutions or stack diffusion sweeps *m*. Kernel's spatial (top row) and frequency (bottom row) responses. This kernel is not explicitly used in the algorithm, but is an implicit feature of the TD framework. “Relative slice index” is the slice index with respect to the slice the kernel is centered at. Kernels were computed for α=0.5 (blue line), α=0.49 (green line) and α=0.45 (red line). For α=0.5 the kernel features a high-pass component that does not attenuate with increasing *m*. For *α* < 0.5, *Ψ* → *G*(*r, t*), as *m* → ∞. The high-pass component attenuates faster for smaller *α*. For α=0.45 amplitude is < 8% for m=10, and <1% for m=20. (For interpretation of the references to colour in this figure legend, the reader is referred to the web version of this article.)

**Algorithm 1:** Transformation Diffusion Reconstruction (TDR) for transformations with closed inverse and composition.

1) Let *m* ← 0.
**Registration sweep:**
2) Register each slice $I_{i},\;i=0,\ldots ,N-1$ onto its two adjacent neighbors to obtain $f_{i,i-1}^{0},f_{i,i+1}^{0}$. For transformations where $f_{i,j}={\left(f_{j,i}\right)}^{-1}$ can be directly computed, only N−1 registrations are necessary; alternatively, 2(N−1) are needed.
**Transformation space sweeps:**
3) For m=1 to M−1 sweeps a. *Update slice transformation* applying (12) $\begin{array}{c} f_{i}^{m,m+1}=\left\{ \begin{array}{cc} \alpha ⊙(f_{i,i-1}^{m}⊕f_{i,i+1}^{m}), & 1\leq i\leq N-2\\ \alpha ⊙(f_{0,1}^{m}⊕f_{0,1}^{m}), & i=0\\ \alpha ⊙(f_{N-1,N-2}^{m}⊕f_{N-1,N-2}^{m}), & i=N-1 \end{array}\right. \end{array}\;\;\;\;\left(18\right)$ b. *Update neighbors transformations* applying (15) $\begin{array}{c} f_{i,i-1}^{m+1}=f_{i-1}^{m,m+1}\circ f_{i,i-1}^{m}\circ {\left(f_{i}^{m,m+1}\right)}^{-1},1\leq i\leq N-1\\ f_{i,i+1}^{m+1}=f_{i+1}^{m,m+1}\circ f_{i,i+1}^{m}\circ {\left(f_{i}^{m,m+1}\right)}^{-1},0\leq i\leq N-2 \end{array}\;\;\;\;\;\;\;\left(19\right)$
**Output composition:**
4) *Correct slices with accumulated transformations*, applying (16) $\begin{array}{c} {\hat{I}}_{i}=\left(f_{i}^{m-1,m}\circ \ldots \circ f_{i}^{0,1}\right)\circ I_{i},i=0,\ldots ,N-1. \end{array}\;\;\;\;\;\;\;\;\left(20\right)$

**Algorithm 2:** Approximated Transformation Diffusion Reconstruction (ATDR) for B-spline transformations in tensor-product form.

1) Let *m* ← 1.
**Registration sweep with coefficient adjustment:**
2) Register each slice $I_{i},\;i=0,\ldots ,N-1$ onto its two adjacent neighbors to obtain $\Delta c_{i,i-1}^{m},\;\Delta c_{i,i+1}^{m}$. This requires 2(N−1) registrations.
3) Compute adjusted coefficients $\Delta c_{i,i-1}^{\prime m},\Delta c_{i,i+1}^{\prime \;m}$ with (32) $\begin{array}{c} \Delta {c^{\prime }}_{i,j}=\frac{\Delta c_{i,j}-\Delta c_{j,i}}{2}\\ \Delta {c^{\prime }}_{j,i}=\frac{-\Delta c_{i,j}+\Delta c_{j,i}}{2} \end{array}\;\;\;\;\;\;\;\;\;\;\;\;\;\left(33\right)$
**Transformation space sweeps:**
4) For m=2 to *M* sweeps a. Update slice transformation applying (23) $\begin{array}{c} \Delta c_{i}^{\prime m,m+1}=\left\{ \begin{array}{cc} \alpha (\Delta c_{i,i-1}^{\prime m}+\Delta c_{i,i+1}^{\prime m}), & 1\leq i\leq N-2\\ 2\alpha \Delta c_{0,1}^{\prime \;m} & i=0\\ 2\alpha \Delta c_{N-1,N-2}^{\prime \;m} & i=N-1 \end{array}\right.. \end{array}\;\;\;\;\;\;\;\left(34\right)$ b. Find coefficients $\Delta c_{i,kl}\prime \;m+1$ that fulfill neither injectivity condition (C.1) nor (C.2) c. Restrict update $\Delta c_{i,kl}^{\prime \;m,m+1}$ of each offending coefficient $\Delta c_{i,kl}^{\prime \;m+1}$ to enforce (C.2) d. *Update neighbors transformations* applying (24) $\begin{array}{c} \Delta c_{i,i-1}^{\prime m+1}=\Delta c_{i-1}^{\prime m,m+1}+\Delta c_{i,i-1}^{\prime \;m}-\Delta c_{i}^{\prime m,m+1},1\leq i\leq N-1\\ \Delta c_{i,i+1}^{\prime m+1}=\Delta c_{i+1}^{\prime \;m,m+1}+\Delta c_{i,i+1}^{\prime \;m}-\Delta c_{i}^{\prime \;m,m+1},0\leq i\leq N-2 \end{array}\;\;\;\;\;\;\;\;\;\;\left(35\right)$
**Output composition:**
5) Correct slices with accumulated transformations, applying (25) $\begin{array}{c} {\hat{I}}_{i}=\left(f_{i}^{m-1,m}+\ldots +f_{i}^{0,1}\right)\circ I_{i},i=0,\ldots ,N-1. \end{array}\;\;\;\;\;\;\left(36\right)$

## Appendices

### A. Diffusion step *α*

In this appendix we provide a theoretical analysis for the choice of the value of parameter *α*, the diffusion step in TDR and ATDR. First, we solve for $\phi_{i}^{m+1}$ in (5)

(A.1) $$\phi_{i}^{m+1}=\alpha \phi_{i+1}^{m}+\left(1-2\alpha \right)\phi_{i}^{m}+\alpha \phi_{i-1}^{m}.$$

This common solution to the heat equation is not useful for reconstruction because the *ϕ_i_* are unknown, but it is useful for the analysis of *α*. Let $\phi =[\phi_{0},\ldots ,\phi_{N-1}]$, for simplicity omitting the “ghost” slices created by the Neumann boundary conditions. Then, $\phi^{m+1}$ can be computed by discrete convolution ⋆ as

(A.2) $$\begin{array}{c} \phi^{m+1}=\phi^{m}☆\psi \\ \psi =\left[\begin{array}{ccc} \alpha , & \left(1-2\alpha \right), & \alpha \end{array}\right]. \end{array}$$

By linearity, this is equivalent to filtering the initial solution with a kernel formed by repeated convolution

(A.3) $$\phi^{m+1}=\phi^{0}☆\left(\underset{m+1}{\underset{︸}{\psi ☆\ldots ☆\psi }}\right)=\phi^{0}☆\Psi$$

Note that this is an implicit feature of the TD framework. The kernel *Ψ* cannot be applied to the stack as *ϕ*^0^ is unknown. As the number of slices and repeated convolutions increase, by the Central Limit Theorem (Yarlagadda, 2010), *Ψ* → *G*(*r, t*), where *G*(*r, t*) is the Gaussian kernel in (3), a low-pass filter that smooths the stack. From (17), the kernel's bandwidth is $\text{FWHM}\propto \sqrt{1/\alpha }$. Hence, the larger the value of *α*, the fewer stack sweeps required, and the optimal value of the diffusion step appears to be α=0.5 However, for a small number of iterations and *α* ≈ 0.5, *Ψ* also presents a high-pass component that preserves high frequency registration noise. As shown in Fig. 10, for α=0.5 the kernel's high frequency component does not attenuate with increasing *m*, and thus high frequency registration noise would be preserved (although the bandwidth of preserved noise would decrease too). For α=0.49 the high-frequency attenuates and is negligible for m=80. However, its amplitude is ≈60%−20% in the range m=10 to 40, which is common for our reconstructions. By contrast, lowering the diffusion step to α=0.45 is a good trade-off, that increases the number of stack sweeps only by 11% but makes the high-frequency component's amplitude < 8%−1% even for m=10 to 20.

### B. Radius-1 neighborhood vs. radius-*d* neighborhood

Let a radius-*d* neighborhood $\left[I_{i-d},\ldots ,I_{i+d}\right]$ be the set of slices that influence the update of slice *I_i_*. As advanced in the introduction, different methods in the literature propose using radius-1 neighborhoods or larger radius-*d* neighborhoods for reconstruction. In this section, we show that *M* sweeps with the translation TD radius-1 neighborhood produce the same result as one sweep with a radius- *M* neighborhood. Let's consider two consecutive slice updates from (10)

(B.1) $$\phi_{i}^{m,m+1}=\alpha \left(\phi_{i,i+1}^{m}+\phi_{i,i-1}^{m}\right),$$

(B.2) $$\phi_{i}^{m+1,m+2}=\alpha \left(\phi_{i,i+1}^{m+1}+\phi_{i,i-1}^{m+1}\right).$$

To simplify the exposition, we omit the end slices. (B.1) and (B.2) smooth each slice with a radius-1 neighborhood, $I_{i-1},I_{i+1}$. Expanding (B.2) with the neighbor transformation update (24), we obtain

(B.3) $$\phi_{i}^{m+1,m+2}=\alpha \left(\phi_{i,i+1}^{m}+\phi_{i,i-1}^{m}\right)+\alpha \left(\phi_{i+1}^{m,m+1}+\phi_{i-1}^{m,m+1}-2\phi_{i}^{m,m+1}\right).$$

Adding $\phi_{i}^{m,m+1}$ to both sides of the equation, and noting that by transitivity a two sweeps update is $\phi_{i}^{m,m+2}=\phi_{i}^{m,m+1}+\phi_{i}^{m+1,m+2}$,

(B.4) $$\begin{array}{ccc} \phi_{i}^{m,m+2} & = & \alpha \left(\phi_{i,i+1}^{m}+\phi_{i,i-1}^{m}\right)+\alpha \left(\phi_{i+1}^{m,m+1}+\phi_{i-1}^{m,m+1}\right)\\ & & +\;\left(1-2\alpha \right)\phi_{i}^{m,m+1}. \end{array}$$

Using (B.1) to expand $\phi_{i+1}^{m,m+1}$ and $\phi_{i-1}^{m,m+1}$, and using $\phi_{i,j}^{m}=-\phi_{j,i}^{m}$

(B.5) $$\begin{array}{ccc} \phi_{i}^{m,m+2} & = & \alpha \left(2-3\alpha \right)\phi_{i,i+1}^{m}+\alpha \left(2-3\alpha \right)\phi_{i,i-1}^{m}\\ & & +\;\alpha^{2}\phi_{i+1,i+2}^{m}+\alpha^{2}\phi_{i-1,i-2}^{m}. \end{array}$$

By the transitive property, $\phi_{i\pm 1,i\pm 2}^{m}=\phi_{i,i\pm 2}^{m}-\phi_{i,i\pm 1}^{m}$. Substituting into (B.5)

(B.6) $$\begin{array}{ccc} \phi_{i}^{m,m+2} & = & \alpha^{2}\phi_{i,i+2}^{m}+2\alpha \left(1-2\alpha \right)\phi_{i,i+1}^{m}\\ & & +\;2\alpha \left(1-2\alpha \right)\phi_{i,i-1}^{m}+\alpha^{2}\phi_{i,i-2}^{m}. \end{array}$$

That is, the two radius-1 sweeps in (B.1) and (B.2) produce the same result as the single radius-2 sweep in (B.5). By induction, *M* radius-1 sweeps produce the same refinement as one radius-*M* sweep (if the corresponding weights are computed). This result requires transitivity, which will be only approximate with real data.

### C. B-spline injectivity conditions

Let $\Delta c_{kl}=\left(\Delta c_{kl,x},\Delta c_{kl,y}\right)$. Choi and Lee's (2000) sufficient conditions for injectivity of the B-spline defined in (31) are

(C.1) $$\Delta c_{kl,x}<1/K_{2}\;\;\text{and}\;\;\Delta c_{kl,y}<1/K_{2},$$

(C.2) $${\left(\Delta c_{kl,x}\right)}^{2}+{\left(\Delta c_{kl,y}\right)}^{2}<{\left(1/A_{2}\right)}^{2},$$

where *K*_2_ ≈ 2.046392675 and $A_{2}=\sqrt{{\left(3/2\right)}^{2}+{\left(K_{2}-3/2\right)}^{2}}$. Fulfilling either condition guarantees local injectivity of the B-spline. These conditions are known to be conservative, and thus act as regularizers that strongly limit B-spline deformations. On the other hand, they can be directly applied to the control points, whereas more recent work that proposes looser bounds (Chun and Fessler, 2009) applies to the edges of the control polygon and thus, are not trivial to apply to the ATDR algorithm.

To guarantee injectivity, after each diffusion iteration we flag control points that fulfill neither condition. We then normalize the displacement of problematic control points to enforce condition (C.2) as $\Delta c_{kl}/\left(|\Delta c_{kl}|\left(A_{2}-ɛ\right)\right)$ , where ɛ is a small constant. We set ɛ=0.01 as suggested by Choi and Lee (2000).

## Appendix A Supplementary materials

Supplementary Data S1 Supplementary Raw Research Data. This is open data under the CC BY license http://creativecommons.org/licenses/by/4.0/ Supplementary Data S1

Supplementary Data S2 Supplementary Raw Research Data. This is open data under the CC BY license http://creativecommons.org/licenses/by/4.0/ Supplementary Data S1

## Footnotes

1 https://github.com/vigente/gerardus/tree/papers/casero2015_3d_histology_diffusion_registration.

2 Karnovsky's fixative was used for MR imaging, unrelated to this work. This hardened the tissue and made it more brittle than if it had been prepared in a standard manner for histology use.
